# Three patients with homozygous familial hypercholesterolemia: Genomic sequencing and kindred analysis

**DOI:** 10.1002/mgg3.1007

**Published:** 2019-10-16

**Authors:** Karen H.Y. Wong, Michal Levy‐Sakin, Walfred Ma, Nina Gonzaludo, Angel C.Y. Mak, Dedeepya Vaka, Annie Poon, Catherine Chu, Richard Lao, Melek Balamir, Zoe Grenville, Nicolas Wong, John P. Kane, Pui‐Yan Kwok, Mary J. Malloy, Clive R. Pullinger

**Affiliations:** ^1^ Cardiovascular Research Institute University of California San Francisco CA USA; ^2^ Lung Biology Center University of California San Francisco CA USA; ^3^ Institute for Human Genetics University of California San Francisco CA USA; ^4^ Department of Internal Medicine Istanbul University Istanbul Turkey; ^5^ Department of Medicine University of California San Francisco CA USA; ^6^ Department of Biochemistry and Biophysics University of California San Francisco CA USA; ^7^ Department of Dermatology University of California San Francisco CA USA; ^8^ Department of Pediatrics University of California San Francisco CA USA; ^9^ Department of Physiological Nursing University of California San Francisco CA USA

**Keywords:** 10xG linked‐reads whole genome sequencing, dyslipidemia, LDL, whole exome sequencing

## Abstract

**Background:**

Homozygous Familial Hypercholesterolemia (HoFH) is an inherited recessive condition associated with extremely high levels of low‐density lipoprotein (LDL) cholesterol in affected individuals. It is usually caused by homozygous or compound heterozygous functional mutations in the LDL receptor (*LDLR*). A number of mutations causing FH have been reported in literature and such genetic heterogeneity presents great challenges for disease diagnosis.

**Objective:**

We aim to determine the likely genetic defects responsible for three cases of pediatric HoFH in two kindreds.

**Methods:**

We applied whole exome sequencing (WES) on the two probands to determine the likely functional variants among candidate FH genes. We additionally applied 10x Genomics (10xG) Linked‐Reads whole genome sequencing (WGS) on one of the kindreds to identify potentially deleterious structural variants (SVs) underlying HoFH. A PCR‐based screening assay was also established to detect the *LDLR* structural variant in a cohort of 641 patients with elevated LDL.

**Results:**

In the Caucasian kindred, the FH homozygosity can be attributed to two compound heterozygous *LDLR* damaging variants, an exon 12 p.G592E missense mutation and a novel 3kb exon 1 deletion. By analyzing the 10xG phased data, we ascertained that this deletion allele was most likely to have originated from a Russian ancestor. In the Mexican kindred, the strikingly elevated LDL cholesterol level can be attributed to a homozygous frameshift *LDLR* variant p.E113fs.

**Conclusions:**

While the application of WES can provide a cost‐effective way of identifying the genetic causes of FH, it often lacks sensitivity for detecting structural variants. Our finding of the *LDLR* exon 1 deletion highlights the broader utility of Linked‐Read WGS in detecting SVs in the clinical setting, especially when HoFH patients remain undiagnosed after WES.

## Graphical abstract

The genetic defects underlying homozygous familial hypercholesterolemia (HoFH) are difficult to ascertain due to its genetic heterogeneity. Here, we applied whole exome and whole genome sequencing with Linked‐Reads on three HoFH patients and they revealed a deleterious missense mutation, a frameshift mutation, and a novel 3kb deletion, all of which are in the LDLR gene. Our study underlines the importance of accurate structural variation detection in clinical sequencing pipelines.

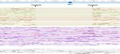

**Abbreviations**
CHD: coronary heart disease
LDL‐C: low‐density lipoprotein cholesterol
MAF: minor allele frequency
TC: total cholesterol
TG: triglyceride

## 1 INTRODUCTION

Mutations in the low‐density lipoprotein (LDL) receptor (*LDLR;* OMIM accession: 606945) underlie most cases of familial hypercholesterolemia (FH). This monogenic disorder represents approximately 4% of patients with plasma levels of LDL cholesterol (LDL‐C) above the 95th percentile and normal levels of other lipoproteins. The prevalence of heterozygous FH (HeFH) within the general population has been traditionally estimated to be in the range of 1 in 400 to 500 (Goldstein, Hobbs, & Brown, 2007), but more contemporary studies suggested that the frequency can be as high as 1 in 200 in the European general population (Benn, Watts, Tybjaerg‐Hansen, & Nordestgaard, 2012, 2016; Nordestgaard et al., 2013). It is typically associated with premature coronary artery disease (CAD) and peripheral vascular disease. Other clinical findings include tendon xanthomas, xanthelasma, and arcus corneae. However, the phenotype of FH can manifest in a much more severe way when individuals harbor homozygous or compound HeFH variants. Individuals with HeFH have a mean LDL‐C plasma level of 298 mg/dl whereas those with HoFH have a mean of 625 mg/dl (Goldstein et al., 2007). Among 65 HeFH patients seen at the UCSF Lipid Clinic with known deleterious *LDLR* mutations, the mean LDL‐C was 288 mg/dl (unpublished observations: CRP, JPK, MJM). Although deleterious mutations in the *LDLR* are the most common causes of severely elevated LDL‐C, mutations in other genes account for a few cases. Among these genes are *APOB* (apolipoprotein B*;* OMIM accession: 107730), coding for a ligand for the LDLR; *PCSK9* (proprotein convertase subtilisin/kexin type 9*;* OMIM accession: 607786); *LDLRAP1* (low‐density lipoprotein receptor adaptor protein 1*;* OMIM accession: 605747), *STAP1* (signal transducing adaptor family member 1*;* OMIM accession: 604298), and *CYP7A1* (cholesterol 7α‐hydroxylase*;* OMIM accession: 118455) (Hegele et al., 2015; Pullinger, Kane, & Malloy, 2003), *LIPA* (lysosomal acid lipase*;* OMIM accession: 613497) (Pullinger et al., 2015), *ABCG5* (ATP binding cassette subfamily G member 5*;* OMIM accession: 605459) and *ABCG8* (ATP binding cassette subfamily G member 8*;* OMIM accession: 605460) (Pullinger et al., 2003).

Establishing a definitive genetic etiology of FH is important because it is useful in guiding physicians in determining the most effective management on the basis of a specific mutation. For example, if a patient is homozygous, or compound heterozygous, for damaging or null *LDLR* variants, statins alone will have limited effect because these drugs work by increasing the LDL receptor expression on the cell surface thereby removing circulating LDL and remnant lipoproteins from the blood. In the case of defects in *ABCG5* and *ABCG8* transporters the dyslipidemia of FH is compounded by hyperabsorption of sterols. Agents with mechanisms of action not dependent on the presence of competent LDL receptors (such as ezetimibe, niacin, bile acid ion‐exchange resins, and lomitapide) are useful. In patients with LIPA deficiency statins are likely to exacerbate the disease by increasing endocytosis of cholesteryl esters.

Here we report two families with three patients with rare HoFH. In the first kindred, one homozygous and seven heterozygous patients were identified. The other kindred included two homozygous and three heterozygous patients.

## 2 METHODS

### 2.1 Study patients

Two probands with HoFH were referred to the UCSF Pediatric Lipid Clinic. The first was a 5‐year old Caucasian girl who presented with a severely elevated level of LDL and tuberous and tendinous xanthomas. The second was a 21‐month old girl of Hispanic ancestry who, when first seen, also had a severely elevated level of LDL‐C. She had some cutaneous xanthomas at birth. For each kindred, blood samples were also obtained from available family members; 11 relatives for the first kindred and four for the second. All study participants gave written informed consent prior to their enrollment in the study, which adhered to the World Medical Association Declaration of Helsinki and were approved by the University of California San Francisco (UCSF) Committee on Human Research Institutional Review Board as part of the UCSF Human Research Protection Program. Children were included with parental consent.

### 2.2 DNA preparation and biochemical analyses

Blood was collected from the two probands and their participating family members, after overnight fasting, in tubes containing 0.1% ethylenediaminetetraacetic acid. When these samples were collected, none of the participants were taking lipid medications. Blood was centrifuged at 3,000 rpm for 20 min at 4°C and plasma separated. An automated chemical analyzer (COBAS Chemistry analyzer) was used to measure levels of total cholesterol (TC), HDL cholesterol (HDL‐C), and triglyceride (TG) in plasma as described previously (Pullinger et al., 2008, 1995). The Friedewald method was used to calculate LDL‐C (Friedewald, Levy, & Fredrickson, 1972). Genomic DNA was extracted using the Wizard purification kit (Qiagen). The Genbank reference sequences used for sanger validations were: *LDLR* NG_009060.1; *APOB* NG_011793.1; *MYL5* NM_002477.1; *MSR1* NG_012102.1; *ABCA1* NG_007981.1; *SPTY2D1* NM_194285.3; *LCAT* NG_009778.1; *PCTP* NM_021213.4; *LPIN1* NG_012843.2; *STAB1* NM_015136.3; *ABCC2* NG_011798.2; *PGS1* NM_024419.5; *OSBPL1A* NG_029432.1; *MC4R* NG_016441.1.

### 2.3 Whole exome sequencing

Genomic DNA derived from the probands of the two kindreds (1ug/sample) was sheared to an average fragment size of 300bp. DNA libraries were prepared using the KAPA DNA library preparation kits for Illumina sequencing platforms. Exons were captured using a Roche NimbleGen SeqCap EZ library probe and the captured libraries were sequenced on a HiSeq2500. Processing of image files was performed using standard protocol. Alignment to Hg19 reference was performed using Burrows–Wheeler Aligner (BWA v0.7.15). Picard v2.5.0 was used to mark duplicate and low‐quality reads. Finally, GATK (Mckenna et al., 2010) was used for variant calling.

### 2.4 WES variant calling pipeline

Resulting variants were annotated with Annovar (Wang, Li, & Hakonarson, 2010) to evaluate their effect on coding sequences, allele frequency in the general population, and the predicted level of pathogenicity. Synonymous, intronic, intergenic, and untranslated region (UTR) variants were removed, along with variants with low read depth support (>20). Variants were intersected with a manually curated list of 594 lipid metabolism candidate genes including those in lipid metabolism pathways and genome‐wide associated study (GWAS) hits (Table S1). Using reported allele frequencies from the GnomAD, TOPMED, and ExAC, common variants with a minor allele frequency >1% were discarded, except for two potentially damaging variants. Minor allele frequencies, prior evidence of disease causality, and pathogenicity scores predicted by SIFT (Ng & Henikoff, 2003) and Polyphen‐2 (Adzhubei, Jordan, & Sunyaev, 2013) were used to prioritize variants during the curation of the filtered variant list. We put emphasis on variants in genes with strong previous clinical and biochemical evidence. We ranked the remaining variants based on SIFT and Polyphen‐2 algorithm scores. The chosen variants were then manually verified using Sanger sequencing.

### 2.5 Final variant assessment

After the above described filtering process, we selected eight potentially damaging variants for each kindred to study further. These 16 variants were examined using Sanger sequencing using DNA from the appropriate family members. Sanger sequencing was performed using BigDye Terminator v3.1 and a 3730xl DNA Analyzer, Applied Biosystems on PCR products generated from primers designed using MacVector software (MacVector, Inc.).

### 2.6 10x Genomics whole genome sequencing

**Figure 1:**
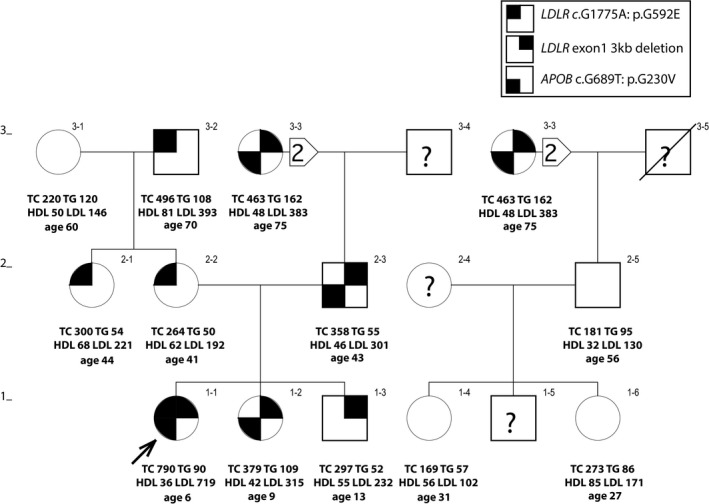
Pedigree of a Caucasian‐American family showing the distribution of the *APOB* (c.G689T: p.G230V) and *LDLR* (c.G1775A: p.G592E and exon1 3kb deletion) mutations. Lipid values are in mg/dl. Ages and body mass indexes are those at time of drawing blood

Following WES, 10xG WGS was also performed on the father of the Caucasian proband (Figure 1; subject 2–3). This was because a fresh blood sample was required here and we wanted to avoid drawing blood again from this child. High molecular weight genomic DNA extraction, sample indexing, and generation of partition barcoded libraries were performed according to 10x Genomics (Pleasanton) Chromium Genome User Guide and as published previously (Weisenfeld, Kumar, Shah, Church, & Jaffe, 2017). Raw reads were processed and aligned to the reference genome using 10x Genomics' Long Ranger software with the “wgs” pipeline with default settings. Deletion coordinates generated by Long Ranger overlapped with the disease relevant gene list and the *LDLR* exon 1 deletion rose to the top of our variant candidate list. The *LDLR* deletion mutation observed here was confirmed by Sanger sequencing using primers spanning the breakpoint: forward: 5'‐agctcctagaacttgcctatcct‐3' and reverse 5'‐gaggctgtctctctgcaactaat‐3'.

### 2.7 LDLR 3kb Exon 1 deletion screening assay

A PCR‐based diagnostic assay was established to detect the presence of the *LDLR* 3kb deletion which eliminates the promoter region as well as exon 1. We followed the approach of Simard et al (Simard et al., 2004) to develop a multiplex PCR assay with two primers flanking the deletion breakpoints (as determined using 10xG WGS) and two primers within the deleted region to detect the presence of a wild‐type allele. The first pair of primers were: 5'‐agctcctagaacttgcctatcct‐3' and 5'‐tcgccacagagcacagcggaa‐3', which generate a 254 bp product from the mutant allele. The second pair of primers were: 5'‐caacaaatcaagtcgcctgcc‐3' and 5'‐tgccattaccccacaagtctc‐3', which yield a 481 bp product from the wild‐type allele. We used this assay to screen all family members in kindred 1, and also a cohort of patients with elevated LDL‐C. In this same cohort, using the previously described method (Simard et al., 2004) we found three patients with the French‐Canadian *LDLR* exon 1 15.9 kb deletion (unpublished observations: CRP, JPK, MJM).

### 2.8 Ancestry determination for the LDLR 3kb Exon 1 deletion

To confirm that the patient was of European descent, we leveraged publicly available single nucleotide polymorphism (SNP) genotyping data from the Human Genome Diversity Project (HGDP; download link: http://hagsc.org/hgdp/data/hgdp.zip ). Tri‐allelic variants, sex chromosome variants, and variants with MAF <0.05 were discarded. Filtered variants were merged with the proband's vcf file followed by LD pruning (plink v1.9 –indep‐pairwise 1,000 5 0.5) using r^2^ = 0.5 as the cutoff. Principal components analysis (PCA) was performed using plink and the top two PCs were used for plotting. Super‐population labels were assigned based on a previous study (Mallick et al., 2016). To ascertain the ancestral origin of the *LDLR* exon 1 deletion, we utilized the phased SNPs generated from 10xG Long Ranger pipeline. We modeled the expected conditional probability of observing the proband's genotype flanking the deletion events given population allele frequencies based on the HGDP dataset (Supplementary Methods).

## 3 RESULTS

### 3.1 Clinical and other characteristics of the patients

#### 3.1.1 Kindred 1

The proband with a clinical diagnosis of HoFH was a 5‐year old Caucasian girl (Figure 1, subject 1–1) who presented in the UCSF Pediatric Lipid Clinic with severely elevated LDL‐C (719 mg/dl), tuberous and tendon xanthomas. Also, her plasma level of HDL cholesterol (HDL‐C) was abnormally low (36 mg/dl). Her father (subject 2–3) and paternal grandmother (subject 3–3) have elevated LDL‐C consistent with HeFH, and each had coronary bypass surgery at ages 43 and 60, respectively. Her mother (subject 2–2), maternal aunt (subject 2–1) and maternal grandfather (subject 3–2) have elevated levels of LDL‐C. The proband has an older sister (subject 1–2) and brother (subject 1–3), both with HeFH.

#### 3.1.2 Kindred 2

**Figure 2:**
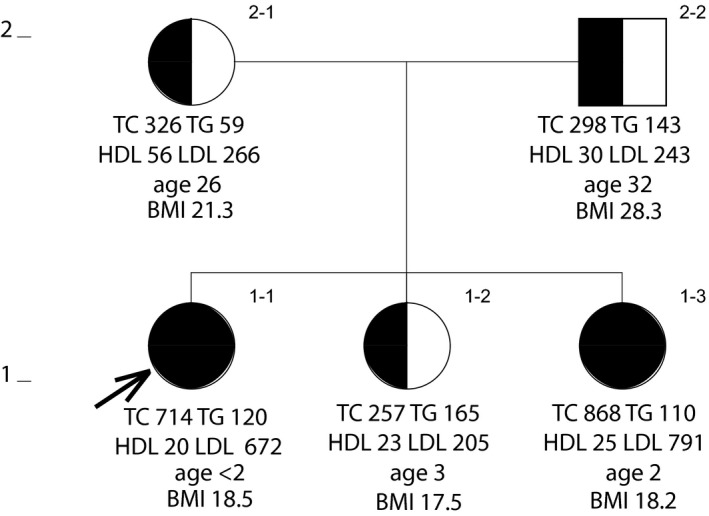
Pedigree of a Mexican‐American family showing the distribution of the *LDLR* frameshift mutation (c.337dupG p.E113fs). Lipid values are in mg/dl. Ages and body mass indexes are those at time of drawing blood

In the kindred of Mexican ancestry, the proband (Figure 2, 1–1) was born with cutaneous xanthomas. She was referred to the UCSF Pediatric Lipid Clinic at age 21 months where a clinical diagnosis of HoFH was made. She had severe hypercholesterolemia with an LDL‐C of 672 mg/dl. By age 5 years her LDL‐C was 925 mg/dl. Her plasma level of HDL cholesterol (HDL‐C) was low (20 mg/dl). A younger sister (subject 1–3) was subsequently seen at the clinic with the same phenotype and more extensive cutaneous xanthomas at birth. At age 7 months her LDL‐C was 791 mg/dl, and HDL‐C was 25 mg/dl. Another sister (subject 1–2) and the parents (subjects 2–1 and 2–2) all presented with a phenotype of HeFH. Two of the proband's grandparents are first cousins (the paternal grandmother is a first cousin of the maternal grandmother).

### 3.2 Whole exome sequencing and whole genome sequencing: kindred 1

**Figure 3:**
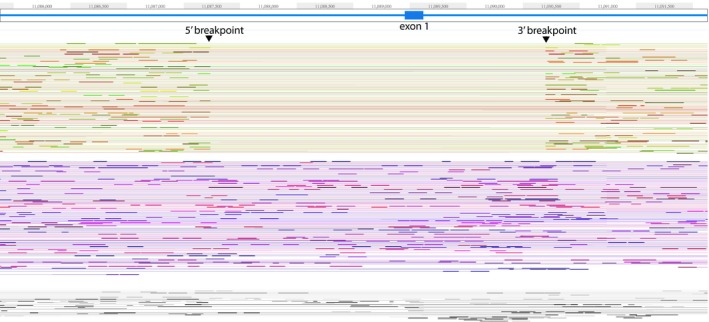
Phased 10x sequencing results from subject 2–3 in kindred 1 showing the breakpoints of the 3kb *LDLR* exon 1 deletion (GRCh38/hg38 coordinates)

**Figure 4:**
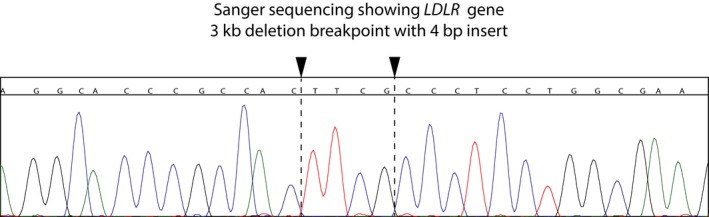
Sanger chromatogram that confirms the breakpoints from 10x sequencing. The 4bp insertion lies between the breakpoints in chromosome 19 at nucleotides 11,198,406 (GRCh37/hg19) and 11,201,384

**Figure 5:**
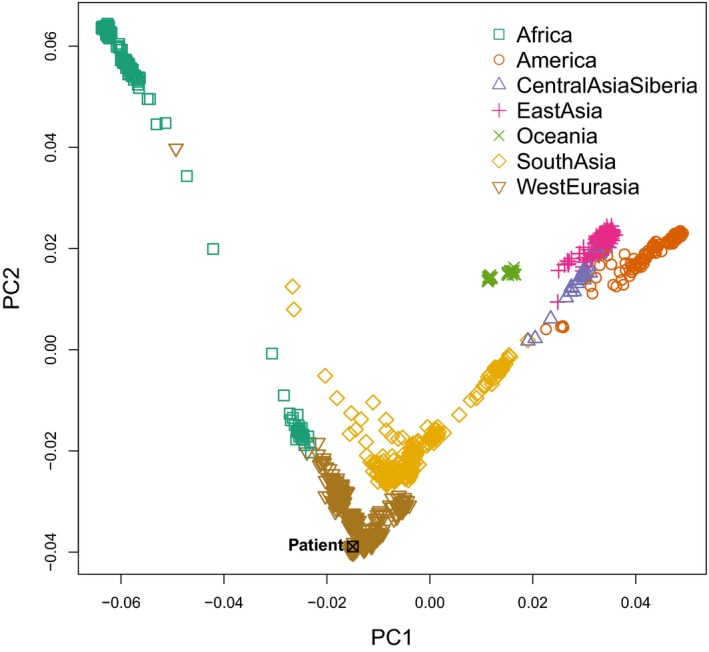
PCA plot illustrating the first two principal components. HGDP samples are used as the reference dataset and the patient from our study is highlighted in black

**Figure 6:**
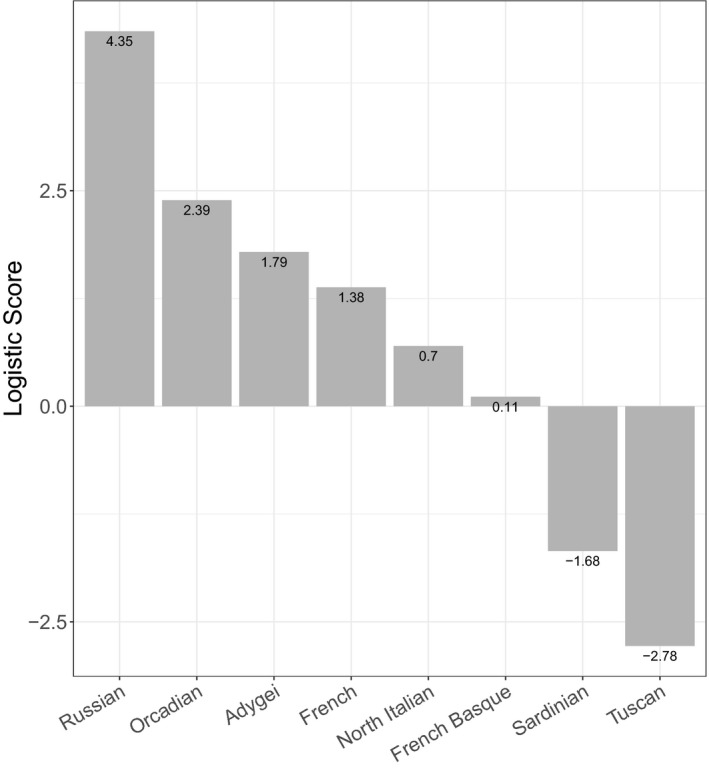
Bar plot showing the distribution of the logistic probability of this entire phased block to be derived from the different European populations

Eight potentially deleterious mutations (Table 1), revealed by WES of DNA from the proband of kindred 1 (subject 1–1; Figure 1), were verified and assessed further by Sanger sequencing using all the available DNA samples from the rest of this family. However, the pattern of inheritance of these eight heterozygous variants did not fully cosegregate with the elevated levels of LDL‐C in this family (Table 2). Notably, subject 1–3, despite his high level of LDL‐C (232 mg/dl), did not carry either of the potentially deleterious *LDLR* (p.G592E) or *APOB* (p.G230V) variants. To comprehensively and robustly evaluate the genome in an unbiased manner, we utilized Linked‐Reads WGS (10x Genomics, Inc.) in an attempt to identify potential disease‐causing structural variants that might have gone undetected by WES. 10xG WGS was chosen because it has been reported that the use of virtual long‐reads allows for a much higher SV detection sensitivity (Wong, Levy‐Sakin, & Kwok, 2018). This revealed a heterozygous 2,977 bp deletion that included all of exon 1 (Figure 3). This mutation was confirmed using Sanger sequencing (Figure 4) of a PCR product using primers spanning the indicated breakpoint. This sequencing revealed an additional four bases (TTCG) between the deletion junction (Figure 4). To the best of our knowledge, this deletion has not been reported previously. Using the genomic information, we validated that this proband of kindred 1 was of European descent (Figure 5). Furthermore, we analyzed the phased SNPs flanking the *LDLR* exon 1 deletion and predicted that this deletion allele was most likely to be originally derived from a Russian ancestor (Figure 6), which is consistent with the patient's self‐reported family migration history.

**Table 1:** Damaging rare mutations found for the proband from kindred 1 using exome sequencing

Chromosome	2	4	8	9	11	16	17	19
Position	21,259,976	673,778	16,001,102	107,589,238	18,637,499	67,976,320	53,844,742	11,227,604
ID	—	rs2228354	—	rs138880920	rs66514853	rs4986970	rs112454522	rs137929307
Gene	APOB	MYL5	MSR1	ABCA1	SPTY2D1	LCAT	PCTP	LDLR
Mutation	exon6: c.G689T: p.G230V	exon4: c.T263C: p.F88S	exon8: c.G998T: p.G333V	exon16: c.G2328C: p.K776N	exon3: c.320_322del AGA p.K107del	exon5: c.T694A: p.S232T	exon2: c.G188A: p.C63Y	exon12: c.G1775A: p.G592E
Clinvar	—	not reported	—	Likely benign	Not reported	—	—	Pathogenic/ Likely pathogenic
GnomAD	—	.00665	—	.0033	.01726	.01758	.00473	.00004
TOPMED	—	.00593	—	.00198	.01695	.01669	.00444	.00003
ExAC	—	.0069	—	.0036	.0169	.01807	.0049	.00005
SIFT	D	D	D	D	—	T	D	D
Polyphen2 HDIV	D	D	D	D	—	D	D	D
Polyphen2 HVAR	D	D	D	D	—	P	D	D
LRT	D	U	D	D	—	U	D	D
Mutation Taster	D	D	D	D	—	D	D	D
Mutation Assessor	M	H	H	M	—	L	M	M
PROVEAN	D	D	D	D	—	*N*	D	D
FATHMM	T	D	D	D	—	D	T	D
GERP_RS	5.64	4.12	4.9	3.27	—	3.84	5.64	5.48
MetaSVM score	−.4609	.7855	1.0032	.3243	—	.1186	−.6575	1.0446
MetaSVM pred	T	D	D	D	—	D	T	D

**Table 2:** Distribution of potentially damaging rare mutations in kindred 1

Gene	APOB	MYL5	MSR1	ABCA1	SPTY2D1	LCAT	PCTP	LDLR	LDLR	LDL‐C	HDL‐C
Mutation	exon6: c.G689T: p.G230V	exon4: c.T263C: p.F88S	exon8: c.G998T: p.G333V	exon16: c.G2328C: p.K776N	exon3: c.320_322del AGA p.K107del	exon5: c.T694A: p.S232T	exon2: c.G188A: p.C63Y	exon 1:2,977 bp del	exon12: c.G1775A: p.G592E		
Method	exome	exome	exome	exome	exome	exome	exome	10x	exome		
Subjects											
1–1	GT	TC	GT	GC	AGA/‐	TA	GA	‐/del	GA	719	36
1–2	GT	TT	GG	GG	AGA/‐	TA	GA	‐/del	GG	315	42
1–3	GG	TT	GG	GG	AGA/‐	TA	GG	‐/del	GG	232	55
1–4	GG	TT	GG	GG	AGA/AGA	TT	GG	‐/‐	GG	102	56
1–6	GG	TT	GG	GG	AGA/AGA	TT	GG	‐/‐	GG	171	85
2–1	GG	TT	GG	GG	AGA/‐	TT	GG	‐/‐	GA	221	68
2–2	GG	TT	GT	GG	AGA/‐	TT	GG	‐/‐	GA	192	62
2–3	GT	TC	GG	GC	AGA/AGA	TA	GA	‐/del	GG	301	46
2–5	GG	TT	GG	GG	AGA/AGA	TT	GG	‐/‐	GG	130	32
3–1	GG	TT	GG	GG	AGA/‐	TT	GG	‐/‐	GG	146	50
3–2	GG	TT	GT	GG	AGA/AGA	TT	GG	‐/‐	GA	393	81
3–3	GT	TT	GG	GC	AGA/AGA	TT	GG	‐/del	GG	383	48

**Figure 7:**
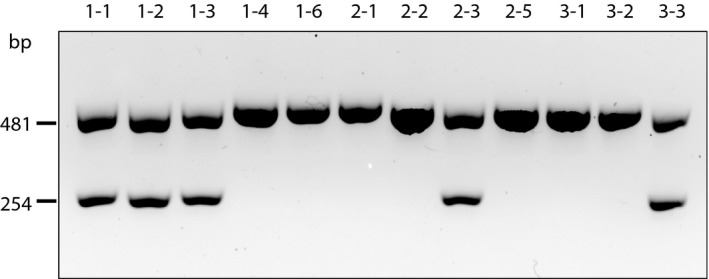
Agarose gel of PCR products showing the carriers in kindred 1 of the *LDLR* exon 1 3kb deletion mutation. The 481 bp control band for the wild‐type allele is from within the deleted region. The 254 bp band is a breakpoint‐spanning band demonstrating the presence of the deletion

To detect this 3kb deletion in other family members, and in a population of patients with elevated levels of LDL‐C, we established a multiplex PCR assay similar in design to that described by Simard et al., (2004). Figure 7 (an agarose gel) shows the pattern of inheritance in kindred 1 with five family members carrying this mutation, including the proband (subject 1–1) and, notably, her brother (subject 1–3). The 481 bp fragment (Figure 7) is located within the deleted region and is a control for the presence of the wild‐type allele, whereas the 254 bp band is a breakpoint‐spanning fragment indicating the presence of the 3kb deletion allele. We subsequently screened 641 unrelated patients who were recruited into the UCSF Genomic Resource in Arteriosclerosis (GRA) (Pullinger et al., 2008; Shiffman et al., 2005), with age and sex‐adjusted levels of LDL‐C above the 95th percentile, but did not detect any additional patients with this exon 1 3kb deletion.

The pattern of inheritance of the two *LDLR* mutations, in addition to the potentially damaging novel *APOB* variant (c.G689T: p.G230V) in kindred 1 is shown in Figure 1 and Table 2. The two *LDLR* mutations now account for the pattern of LDL‐C levels. The degree to which the *APOB* variant contributes an additional adverse effect is not clear, especially as no one in the kindred carries that variant in the absence of a deleterious LDLR mutation.

The proband, in addition to her high level of LDL‐C, has a greatly decreased level of HDL‐C (36 mg/dl); less than the 5th percentile for her age (Lipid Research Clinics Program, 1980). No other member of the kindred has a level of HDL‐C that low. Two of the potentially damaging variants listed in Table 1 are in genes that have been directly associated with low levels of HDL‐C (Lange, Willer, & Rich, 2015). These are *ABCA1* (ATP binding cassette subfamily A member 1) p.K776N and *LCAT* (lecithin‐cholesterol acyltransferase) p.S232T. The first of these substitutions, which occurs in the 5th transmembrane helix of the ABCA1 protein, is a more likely cause of the low HDL‐C based on several damaging predictions (Table 1), and because the residue is conserved. However, two other carriers (subjects 2–3 and 3–3) do not have notably low HDL‐C. Among the other potentially damaging variants, *MYL5* (myosin light chain 5) p.F88S, is in a gene that is one of 10 shown to be regulated by the master trans regulator *KLF14 *(Consortium et al., 2011), itself associated with levels of HDL‐C (Lange et al., 2015) and type 2 diabetes. An additional carrier (subject 2–3), however, has a normal level of HDL‐C.

### 3.3 Exome and other sequencing: kindred 2

Eight potentially deleterious variants detected using exome sequencing of DNA from the proband in kindred 2 (Table 3) were verified, and the rest of the family was screened for them using Sanger sequencing. Table 4 and Figure 2 show the distribution of these variants within the family. *LDLR* mutation (p.E113fs) is the only mutation that is present in the homozygous state (subjects 1–1 and 1–3). None of the other seven seem to contribute to the pattern of elevated LDL‐C.

**Table 3:** Damaging rare mutations found for the proband from kindred 2

Chromosome	2	3	8	10	17	18	18	19
Position	11,919,668	52,556,168	16,012,594	101,594,176	76,395,566	21,957,382	58,038,777	11,215,918
ID	rs141555457	rs566837633	rs41341748	rs142715085	rs780258683	rs766116535	rs79783591	rs752191968
Gene	LPIN1	STAB1	MSR1	ABCC2	PGS1	OSBPL1A	MC4R	LDLR
Mutation	exon6: c.C746A: p.T249K	exon59: c.C6387G: p.S2129R	exon6: c.C877T: p.R293X	exon24: c.C3298A: p.R1100S	exon5: c.G649A: p.G217S	exon2: c.114_115insAATT: p.C39fs	exon1: c.T806A: p.I269N	exon4: c.337dupG: p.E113fs
Clinvar	Not reported	Not reported	Pathogenic; uncertain significance. Hereditary cancer	Uncertain significance	Not reported	Not reported	Likely pathogenic; uncertain significance: Obesity	Pathogenic: Familial hypercholesterolemia
GnomAD	.00054	.00092	.00821	.00048	.00002	.0022	.00103	—
TOPMED	.00017	.00035	.00765	.0007	.00002	.00259	.00026	—
ExAC	.00048	.00081	.0077	.0005	.00002	.00187	.00081	.00001
SIFT	D	D	—	T	D	—	D	—
Polyphen2 HDIV	D	D	—	D	D	—	D	—
Polyphen2 HVAR	P	P	—	D	D	—	D	—
LRT	D	*N*	—	D	D	—	D	—
Mutation Taster	D	D	—	D	D	—	D	—
Mutation Assessor	M	L	—	L	M	—	L	—
PROVEAN	D	D	—	D	T	—	D	—
FATHMM	D	D	—	D	T	—	T	—
GERP_RS	4.85	3.83	2.84	4.38	5.5	—	5.85	—
MetaSVM score	.5039	.4546	—	.5245	−.5799	—	−.7544	—
MetaSVM pred	D	D	—	D	T	—	T	—

**Table 4:** Distribution of rare mutations in kindred 2

Gene	LPIN1	STAB1	MSR1	ABCC2	PGS1	OSBPL1A	MC4R	LDLR	LDL‐C	HDL‐C
Mutation	exon6: c.C746A: p.T249K	exon59: c.C6387G: p.S2129R	exon6: c.C877T: p.R293X	exon24: c.C3298A: p.R1100S	exon5: c.G649A: p.G217S	exon2: c.115_116 insAATT: p.C39X	exon1: c.T806A: p.I269N	exon4: c.337dupG: p.E113fs		
Subject										
1–1	CA	CG	CT	CA	GA	‐/AATT	TA	G/G	672	20
1–2	CA	CG	CT	CC	GG	‐/‐	TT	‐/G	205	23
1–3	CA	CC	CT	CA	GA	‐/‐	TT	G/G	791	25
2–1	CC	CC	CT	CC	GA	‐/AATT	TT	‐/G	266	56
2–2	CA	CG	CC	CA	GG	‐/‐	TA	‐/G	243	30

The pattern of inheritance of seven of the variants does not match the low levels of HDL‐C seen in four of the five family members. Only one does, that of the *LPIN1* (lipin 1) p.T249K mutant. This mutation (rs141555457) is extremely rare, and is predicted to be probably pathogenic, but there is no record in ClinVar. The gene codes for an intracellular phosphatidic acid phosphohydrolase that is an important regulator of lipid metabolism, especially hepatic VLDL‐triglyceride secretion (Chen et al., 2008). Deleterious mutations in *Lpin1* can cause fatty liver dystrophy and various dyslipidemias in mice (Chen et al., 2008). In humans non‐alcoholic fatty liver disease (NAFLD) is often accompanied by low levels of HDL‐C. A heterozygous carrier of a predicted pathogenic mutation in *LPIN1* was responsible for statin‐induced myopathy (Zeharia et al., 2008). It is not clear whether a heterozygous functional variant in *LPIN1* could be the cause of the low levels of HDL‐C seen here.

## 4 DISCUSSION

Here we report three patients with HoFH from two kindreds. The Caucasian kindred had one homozygous and seven heterozygous individuals caused by two separate deleterious *LDLR* variants (exon 1 3kb deletion and p.G592E), with the possibility that a putatively deleterious novel *APOB* variant (p.G230V) contributes to the severity of FH in this family. The novel 3kb deletion originates from the proband's paternal family and was found among five individuals, while the p.G592E mutation is of maternal origin with four carriers. The *LDLR* exon 1 3kb deletion removing all of exon 1 and the proximal promotor is a null mutation, a priori. The p.G592E mutation occurs in exon 12 in a spacer domain within the 400‐amino acid region that contains three cysteine‐rich repeats, each having homology to epidermal growth factor (EGF). This spacer region between EGF repeats 2 and 3 constitutes a six‐bladed beta‐propeller structure with six “YWTD” repeats (Jeon et al., 2001). The p.G592E mutation occurs within the 5th of these repeats at a highly conserved glycine residue (Jeon et al., 2001). This variant has been reported a number of times among Italian, Polish, German and Spanish patients (Bochmann et al., 2001; Górski, Kubalska, Naruszewicz, & Lubiński, 1998; Hobbs, Brown, & Goldstein, 1992; Mozas et al., 2004). With this variant, a class 5 mutation, the receptor has some residual activity (Romano et al., 2010) with the protein product binding and internalizing but failing to release LDL and not recycling to the plasma membrane. The affected residue is highly conserved.

Additionally, in kindred 1 there were three other potentially deleterious variants that might affect levels of LDL‐C. One was the novel p.G333V variant in *MSR1* (macrophage scavenger receptor 1). It encodes for three types of class A macrophage scavenger receptors via alternate splicing. These bind, among numerous other ligands, modified LDL particles. The second variant, p.C63Y, was in *PCTP* (phosphatidylcholine transfer protein). Genetic studies indicate that the PCTP protein plays a role in HDL and VLDL metabolism and can affect LDL particle size (Dolley et al., 2007). The pattern of inheritance of these two variants seems to suggest that they cannot account significantly for the elevated level of LDL‐C. *SPT2D1* (SPT2 chromatin protein domain containing 1) has been widely reported to be associated with plasma levels of total cholesterol (Asselbergs et al., 2012; Lange et al., 2015; Teslovich et al., 2010). The p.K107del, in this gene seems unlikely to be causative here because one carrier (subject 3–1) has a level of LDL‐C in the normal range.

The proband in kindred 1 had a significantly decreased level of HDL‐C. She is a carrier of the *ABCA1* p.K776N variant, which at first sight appears as a possible cause here. However, a previous study showed that female carriers did not have lower levels of HDL‐C compared to noncarriers (Frikke‐Schmidt, Nordestgaard, Schnohr, Steffensen, & Tybjaerg‐Hansen, 2005). Another report claimed that it did not demonstrate an “unequivocal segregation” in a family study (Alrasadi, Ruel, Marcil, & Genest, 2006). ClinVar reports it to be “likely benign”. Also in kindred 1 there are two other carriers of this p.K776N variant, and neither has a notably low level of HDL‐C.

*LCAT* (lecithin‐cholesterol acyltransferase) is another candidate gene associated with levels of HDL (Lange et al., 2015). The *LCAT* p.S232T variant has a MAF of 0.0176, making it not so uncommon in the human population. In a study of those with either high (>95th percentile) or low HDL‐C (<5th percentile), the p.S232T variant was found only among those with high HDL‐C (Naseri, Hedayati, Daneshpour, Bandarian, & Azizi, 2014). In kindred 1 it does not show segregation with levels of HDL‐C. Likewise, another potential cause of low HDL‐C, p.F88S variant in *MYL5* (regulated by *KLF14)*, can probably be ruled out because the father has a normal level of HDL‐C and carries this variant as well. Unless there are other undetected damaging mutations, it is likely that the low HDL‐C observed in the proband is a consequence of the extremely high level of LDL‐C. FH heterozygotes and homozygotes tend to have low levels of HDL with homozygotes having the lowest levels, although reason for this is unknown (Goldstein et al., 2007).

In the second kindred, the single *LDLR* frameshift mutant (p.E113fs) is sufficient to explain the strikingly elevated levels of LDL‐C. This extremely rare, a priori, damaging variant has a pathogenic ClinVar entry. It has been previously reported among Mexican FH heterozygotes (Robles‐Osorio et al., 2006). To our knowledge, the two homozygous cases that we report here are the first such cases to be described. Unlike the first kindred, the presence of low HDL‐C in the proband of kindred 2 does not seem to be explained simply as a result of the high levels of LDL‐C. The *OSBPL1A* (oxysterol‐binding protein‐like protein 1a) variant, p.C39fs (rs766116535) has previously been reported to be associated with decreased levels of HDL‐C (Motazacker et al., 2013, 2016). This gene encodes intracellular oxysterol‐binding proteins that act as lipid (sterols and anionic phospholipids) receptors involved in lipid transfer and signaling. Table 4 clearly shows that the *OSBPL1A* p.C39fs does not explain the very low levels of HDL‐C seen in four members of this family, with the second carrier (subject 2–1) of this variant having a normal level (56 mg/dl).

Without some other cause for the extremely low levels of HDL‐C, we believe that the *LPIN1* variant (p.T249K) is the most likely suspect.

It is important to underline that the clinical management of HoFH patient is challenging, and knowing the underlying genetic defects has significant prognostic and therapeutic implications. Although the application of next‐generation sequencing in clinical settings is becoming more accessible, the number of negative diagnostic results remains high. Here, we were able to pinpoint a clinically relevant structural variant using Linked‐Reads in kindred 1. While analyzing SVs in the clinical context is still in its nascent state, the availability of sequencing technology using long‐reads or virtual long‐reads allows for much better detection sensitivity for SVs, thus expanding the search space for disease‐causing variants that have been previously invisible to geneticists. Subject to consideration of benefit versus cost, the broad implementation of more advanced WGS technology may improve diagnoses for patients with HoFH.

## CONFLICT OF INTEREST

The authors have no relevant disclosures.

## AUTHOR CONTRIBUTIONS

John Kane and Mary Malloy recruited the study participants and conducted the clinical evaluations. Clive Pullinger, Karen Wong, Mary Malloy, John Kane, Michal Sakin and Pui‐Yan Kwok planned and organized the study and wrote the manuscript. Karen Wong, Clive Pullinger, Michal Sakin, John Kane, Mary Malloy and Pui‐Yan Kwok assisted in editing and revision. Wangfei Ma, Nina Gonzaludo, Angel C.Y. Mak, Dedeepya Vaka, Annie Poon, Catherine Chu, Richard Lao, Melek Balamir, Zoe Grenville, and Nicolas Wong were responsible for the acquisition, analysis, and interpretation of data. All authors have approved the final article.

## Data Availability

Patient data are not publicly available due to HIPAA (Health Insurance Portability and Accountability Act of 1996) regulations.
