# Supplementary material for: Three patients with homozygous familial hypercholesterolemia: Genomic sequencing and kindred analysis
Source: Mol Genet Genomic Med. 2019 Oct 16;7(12):e1007. doi: 10.1002/mgg3.1007 (PMC6900368; doi:10.1002/mgg3.1007)
Supplement: Supplementary file 1 [file MGG3-7-e1007-s001.docx]

Supplementary Table 1. A list of 594 lipid metabolism-related candidate genes/loci.

| **Gene** | **NCBI ID** | **Chr** | **Start** | **Stop** | **Kathiresan**  **2008** | **Willer**  **2008** | **Teslovich**  **2010** | **Small**  **2011** | **Brouwers**  **2012** | **Lange**  **2015** | **NCBI*** | **Other** |
| --- | --- | --- | --- | --- | --- | --- | --- | --- | --- | --- | --- | --- |
| A2M | 2 | 12 | 9220304 | 9268558 |  |  |  |  |  |  | + |  |
| A2ML1 | 144568 | 12 | 8822472 | 8887202 |  |  |  |  |  | + |  |  |
| ABCA1 | 19 | 9 | 107543283 | 107690436 | + | + |  |  |  |  | + |  |
| ABCA7 | 10347 | 19 | 1040102 | 1065571 |  |  |  | + |  |  |  |  |
| ABCA8 | 10351 | 17 | 66863430 | 66951533 |  |  | + |  |  |  |  |  |
| ABCB1 | 5243 | 7 | 87132948 | 87342564 |  |  |  |  |  |  | + |  |
| ABCB11 | 8647 | 2 | 169779449 | 169887833 |  |  |  |  |  |  | + |  |
| ABCB4 | 5244 | 7 | 87031361 | 87105019 |  |  |  |  |  |  | + |  |
| ABCC1 | 4363 | 16 | 16043434 | 16236931 |  |  |  |  |  |  | + |  |
| ABCC2 | 1244 | 10 | 101542463 | 101611662 |  |  |  |  |  |  | + |  |
| ABCC3 | 8714 | 17 | 48712218 | 48769063 |  |  |  |  |  |  | + |  |
| ABCD1 | 215 | X | 152990323 | 153010216 |  |  |  |  |  |  | + |  |
| ABCD2 | 225 | 12 | 39945022 | 40013843 |  |  |  |  |  |  | + |  |
| ABCD3 | 5825 | 1 | 94883933 | 94984219 |  |  |  |  |  |  | + |  |
| ABCG1 | 9619 | 21 | 43619799 | 43717354 |  |  |  |  |  |  | + |  |
| ABCG5 | 64240 | 2 | 44039611 | 44065958 |  |  |  |  |  |  | + |  |
| ABCG8 | 64241 | 2 | 44066103 | 44105605 |  |  |  |  |  |  | + |  |
| ABHD5 | 51099 | 3 | 43732375 | 43764217 |  |  |  |  |  |  | + |  |
| ABO | 28 | 9 | 136130563 | 136150630 |  |  |  |  |  |  |  | + |
| ACAA1 | 30 | 3 | 38164201 | 38178733 |  |  |  |  |  |  | + |  |
| ACAA2 | 10449 | 18 | 47309874 | 47340251 | + |  |  |  |  |  |  |  |
| ACACA | 31 | 17 | 35441927 | 35766902 |  |  |  |  |  |  | + |  |
| ACACB | 32 | 12 | 109577202 | 109706031 |  |  |  |  |  |  | + |  |
| ACAD11 | 84129 | 3 | 132558138 | 132660131, |  |  |  |  |  | + |  |  |
| ACADL | 33 | 2 | 211052714 | 211090215 |  |  |  |  |  |  | + |  |
| ACADM | 34 | 1 | 76190043 | 76229355 |  |  |  |  |  |  | + |  |
| ACADS | 35 | 12 | 121163571 | 121177811 |  |  |  |  |  |  | + |  |
| ACADVL | 37 | 17 | 7123153 | 7128585 |  |  |  |  |  |  | + |  |
| ACAT1 | 38 | 11 | 107992258 | 108018895 |  |  |  |  |  |  | + |  |
| ACAT2 | 39 | 6 | 160182989 | 160200087 |  |  |  |  |  |  | + |  |
| ACER1 | 125981 | 19 | 6306510 | 6333640 |  |  |  |  |  |  | + |  |
| ACER2 | 340485 | 9 | 19408925 | 19452500 |  |  |  |  |  |  | + |  |
| ACER3 | 55331 | 11 | 76571917 | 76734850 |  |  |  |  |  |  | + |  |
| ACLY | 47 | 17 | 40023179 | 40075272 |  |  |  |  |  |  | + |  |
| ACOT8 | 10005 | 20 | 44470361 | 44486035 |  |  |  |  |  |  | + |  |
| ACOX1 | 51 | 17 | 73937588 | 73975515 |  |  |  |  |  |  | + |  |
| ACOX2 | 8309 | 3 | 58490863 | 58522929 |  |  |  |  |  |  | + |  |
| ACOX3 | 8310 | 4 | 8368009 | 8442452 |  |  |  |  |  |  | + |  |
| ACSL1 | 2180 | 4 | 185676749 | 185747215 |  |  |  |  |  |  | + |  |
| ACSL3 | 2181 | 2 | 223725732 | 223808119 |  |  |  |  |  |  | + |  |
| ACSL4 | 2182 | X | 108884564 | 108976621 |  |  |  |  |  |  | + |  |
| ACSL5 | 51703 | 10 | 114133916 | 114188138 |  |  |  |  |  |  | + |  |
| ACSL6 | 23305 | 5 | 131289152 | 131347349 |  |  |  |  |  |  | + |  |
| ADAM28 | 10863 | 8 | 24151580 | 24212726 |  |  |  | + |  |  |  |  |
| ADD1 | 118 | 4 | 2843727 | 2930076 |  |  |  |  | + |  |  |  |
| ADH5 | 128 | 4 | 99070978 | 99088788 |  |  |  |  |  | + |  |  |
| ADIPOR2 | 79602 | 12 | 1800247 | 1897845 |  |  |  | + |  |  |  |  |
| AGPAT1 | 10554 | 6 | 32135983 | 32145888 |  |  |  |  |  |  | + |  |
| AGPAT2 | 10555 | 9 | 139567595 | 139581911 |  |  |  |  |  |  | + |  |
| AGPAT3 | 56894 | 21 | 45285116 | 45407475 |  |  |  |  |  |  | + |  |
| AGPAT4 | 56895 | 6 | 161551057 | 161695107 |  |  |  |  |  |  | + |  |
| AGPAT5 | 55326 | 8 | 6565878 | 6619024 |  |  |  |  |  |  | + |  |
| AGPAT6 | 137964 | 8 | 41435707 | 41482520 |  |  |  |  |  |  | + |  |
| AGPAT9 | 84803 | 4 | 84457653 | 84527026 |  |  |  |  |  |  | + |  |
| AGPS | 8540 | 2 | 178257471 | 178408564 |  |  |  |  |  |  | + |  |
| AGT | 183 | 1 | 230838269 | 230850336 |  |  |  |  |  |  | + |  |
| AKR1B1 | 231 | 7 | 134127107 | 134143888 |  |  |  |  |  |  | + |  |
| AKR1C4 | 1109 | 10 | 5238798 | 5260912 |  |  |  |  |  |  | + |  |
| AKR1D1 | 6718 | 7 | 137761178 | 137803050 |  |  |  |  |  |  | + |  |
| AKT1 | 207 | 14 | 104769349 | 104795743 |  |  |  |  |  | + |  |  |
| ALB | 213 | 4 | 74269972 | 74287129 |  |  |  |  |  |  | + |  |
| ALOX5 | 240 | 10 | 45869629 | 45941565 |  |  |  |  |  |  | + |  |
| AMACR | 23600 | 5 | 33987091 | 34008220 |  |  |  |  |  |  | + |  |
| AMPD3 | 272 | 11 | 10471868 | 10529126 |  |  |  |  |  | + |  |  |
| ANGPTL1 | 9068 | 1 | 178849535 | 178871353 |  |  |  |  |  | + |  |  |
| ANGPTL3 | 27329 | 1 | 63063187 | 63071180 | + | + |  |  |  |  |  |  |
| ANGPTL4 | 51129 | 19 | 8429011 | 8439257 |  |  |  |  |  |  | + |  |
| ANGPTL8 | 55908 | 19 | 11239619 | 11241943 |  |  | + |  |  | + |  |  |
| ANKRD1 | 27063 | 10 | 92671857 | 92681032 |  |  |  |  |  |  | + |  |
| ANXA9 | 8416 | 1 | 150982023 | 150995638 |  |  |  |  |  | + |  |  |
| APH1B | 83464 | 15 | 63569749 | 63601325 |  |  |  | + |  |  |  |  |
| APOA1 | 335 | 11 | 116706469 | 116708338 | + | + |  |  |  |  | + |  |
| APOA2 | 336 | 1 | 161192083 | 161193418 |  |  |  |  |  |  | + |  |
| APOA4 | 337 | 11 | 116691418 | 116694011 |  | + |  |  |  |  | + |  |
| APOA5 | 116519 | 11 | 116660086 | 116663136 |  | + |  |  |  |  | + |  |
| APOB | 338 | 2 | 21224301 | 21266945 | + | + |  |  |  |  | + |  |
| APOBEC1 | 339 | 12 | 7649400 | 7670599 |  |  |  |  | + |  |  |  |
| APOBR | 55911 | 16 | 28505993 | 28510282 |  |  |  |  |  |  |  | + |
| APOC1 | 341 | 19 | 45417921 | 45422606 |  | + |  |  |  |  | + |  |
| APOC2 | 344 | 19 | 45449243 | 45452818 |  | + |  |  |  |  | + |  |
| APOC3 | 345 | 11 | 116700624 | 116703787 |  | + |  |  |  |  | + |  |
| APOC4 | 346 | 19 | 45445495 | 45448751 |  | + |  |  |  |  |  |  |
| APOD | 347 | 3 | 195295573 | 195311076 |  |  |  |  |  |  |  | + |
| APOE | 348 | 19 | 45409039 | 45412650 |  | + |  |  |  |  | + |  |
| APOF | 319 | 12 | 56754355 | 56756583 |  |  |  | + |  |  |  |  |
| APOH | 350 | 17 | 64208147 | 64225556 |  |  |  |  |  |  |  | + |
| APOM | 55937 | 6 | 31623671 | 31625987 |  |  |  | + |  |  |  |  |
| ARL15 | 54622 | 5 | 53180614 | 53606403 |  |  | + |  |  | + |  |  |
| ARSD | 414 | X | 2822011 | 2847392 |  |  |  | + |  |  |  |  |
| ASAP3 | 55616 | 1 | 23428563 | 23484631 |  |  |  |  |  | + |  |  |
| ATF6 | 22926 | 1 | 161766244 | 161964070 |  |  |  |  | + |  |  |  |
| ATG4C | 84938 | 1 | 63249803 | 63330050 | + |  |  |  |  |  |  |  |
| ATG5 | 9474 | 6 | 106632352 | 106773695 |  |  |  |  |  | + |  |  |
| ATG7 | 10533 | 3 | 11272324 | 11564704 |  |  |  |  |  | + |  |  |
| B3GALT4 | 8705 | 6 | 33244917 | 33246602 |  | + |  |  |  |  |  |  |
| BAAT | 570 | 9 | 104122699 | 104147287 |  |  |  |  |  |  | + |  |
| BCL7B | 9275 | 7 | 72950686 | 72972024 | + |  |  |  |  |  |  |  |
| BCMO1 | 53630 | 16 | 81272296 | 81324747 |  |  |  |  |  |  | + |  |
| BDH1 | 622 | 3 | 197236654 | 197300194 |  |  |  |  |  |  | + |  |
| BMP1 | 649 | 8 | 22022653 | 22069839 |  |  |  |  |  |  | + |  |
| BRAP | 8315 | 12 | 112079950 | 112123790 |  |  | + |  |  |  |  |  |
| BRCA2 | 675 | 13 | 32315480 | 32399672 |  |  |  |  |  | + |  |  |
| BUD13 | 84811 | 11 | 116618886 | 116643714 | + |  |  |  |  |  |  |  |
| C5AR2 | 27202 | 19 | 47332147 | 47342015 |  |  |  |  | + |  |  |  |
| C6orf106 | 64771 | 6 | 34555065 | 34664625 |  |  | + |  |  |  |  |  |
| C8orf82 | 414919 | 8 | 145751603 | 145754458 |  |  |  | + |  |  |  |  |
| CACNG5 | 27091 | 17 | 64873451 | 64881357 |  |  |  | + |  |  |  |  |
| CAPN3 | 825 | 15 | 42651698 | 42704515 |  |  | + |  |  |  |  |  |
| CARM1 | 10498 | 19 | 10982253 | 11033448 |  |  |  |  |  |  | + |  |
| CAV1 | 857 | 7 | 116164839 | 116201239 |  |  |  |  |  |  | + |  |
| CAV2 | 858 | 7 | 116139444 | 116148595 |  |  |  | + |  |  |  |  |
| CD36 | 948 | 7 | 80231504 | 80308593 |  |  |  |  |  |  | + |  |
| CD93 | 22918 | 20 | 23059993 | 23066977 |  |  |  | + |  |  |  |  |
| CEL | 1056 | 9 | 135937365 | 135947248 |  |  |  |  |  |  | + |  |
| CELSR2 | 1952 | 1 | 109792641 | 109818378 | + | + |  |  |  |  |  |  |
| CERS2 | 29956 | 1 | 150965173 | 150975003 |  |  |  |  |  | + |  |  |
| CERS4 | 79603 | 19 | 8274217 | 8327305 |  |  |  |  | + |  |  |  |
| CETP | 1071 | 16 | 56995835 | 57017756 | + | + |  |  |  |  | + |  |
| CH25H | 9023 | 10 | 90965694 | 90967071 |  |  |  |  |  |  | + |  |
| CHD9 | 80205 | 16 | 53088945 | 53361414 |  |  |  |  |  |  | + |  |
| CILP2 | 148113 | 19 | 19649074 | 19657468 | + | + |  |  |  |  |  |  |
| CITED2 | 10370 | 6 | 139693392 | 139695787 |  |  | + |  |  | + |  |  |
| CLPS | 1208 | 6 | 35762760 | 35765102 |  |  |  |  |  |  | + |  |
| CLU | 1191 | 8 | 27454434 | 27472328 |  |  |  |  |  |  |  | + |
| CMIP | 80790 | 16 | 81478775 | 81745367 |  |  | + |  |  |  |  |  |
| CMTM5 | 116173 | 14 | 23376439 | 23379772 |  |  |  |  |  | + |  |  |
| COBLL1 | 22837 | 2 | 165541256 | 165697928 |  |  | + |  |  | + |  |  |
| COL18A1 | 80781 | 21 | 45405137 | 45513720 |  |  |  |  |  | + |  |  |
| COL4A3BP | 10087 | 5 | 74666928 | 74807806 |  |  |  |  |  |  | + |  |
| CPS1 | 1373 | 2 | 210477682 | 210679107 |  |  |  |  |  | + |  |  |
| CPT1A | 1374 | 11 | 68522088 | 68609399 |  |  |  |  |  |  | + |  |
| CPT1B | 1375 | 22 | 51007290 | 51017096 |  |  |  |  |  |  | + |  |
| CPT2 | 1376 | 1 | 53662101 | 53679869 |  |  |  |  |  |  | + |  |
| CRABP2 | 1382 | 1 | 156699606 | 156713174 |  |  |  |  | + |  |  |  |
| CRAT | 1384 | 9 | 131857073 | 131873070 |  |  |  |  |  |  | + |  |
| CREB3L3 | 84699 | 19 | 4153598 | 4173051 |  |  |  |  |  |  |  |  |
| CREBBP | 1387 | 16 | 3775055 | 3930121 |  |  |  |  |  |  | + |  |
| CROT | 54677 | 7 | 86974951 | 87029112 |  |  |  |  |  |  | + |  |
| CSNK1G2 | 1455 | 19 | 1941161 | 1981337 |  |  |  |  |  |  | + |  |
| CSNK1G3 | 1456 | 5 | 123512099 | 123617045 |  |  |  |  |  | + |  |  |
| CTF1 | 1489 | 16 | 30907928 | 30914881 |  |  | + |  |  |  |  |  |
| CTGF | 1490 | 6 | 132269316 | 132272518 |  |  |  |  |  |  | + |  |
| CUBN | 8029 | 10 | 16865965 | 17171816 |  |  |  |  |  |  | + |  |
| CYP11A1 | 1583 | 15 | 74630103 | 74660081 |  |  |  |  |  |  | + |  |
| CYP11B1 | 1584 | 8 | 143953773 | 143961236 |  |  |  |  |  |  | + |  |
| CYP11B2 | 1585 | 8 | 143991975 | 143999259 |  |  |  |  |  |  | + |  |
| CYP17A1 | 1586 | 10 | 104590288 | 104597290 |  |  |  |  |  |  | + |  |
| CYP19A1 | 1588 | 15 | 51500254 | 51630795 |  |  |  |  |  |  | + |  |
| CYP1A1 | 1543 | 15 | 75011883 | 75017877 |  |  |  |  |  |  | + |  |
| CYP1A2 | 1544 | 15 | 75041184 | 75048941 |  |  |  |  |  |  | + |  |
| CYP21A2 | 1589 | 6 | 32006082 | 32009419 |  |  |  |  |  |  | + |  |
| CYP24A1 | 1591 | 20 | 52769988 | 52790516 |  |  |  |  |  |  | + |  |
| CYP26A1 | 1592 | 10 | 94833232 | 94837641 |  |  |  |  |  |  | + |  |
| CYP27A1 | 1593 | 2 | 219646472 | 219680016 |  |  |  |  |  |  | + |  |
| CYP27B1 | 1594 | 12 | 58156117 | 58160976 |  |  |  |  |  |  | + |  |
| CYP2B6 | 1555 | 19 | 41497204 | 41524301 |  |  |  |  |  |  | + |  |
| CYP2C9 | 1559 | 10 | 96698415 | 96749148 |  |  |  |  |  |  | + |  |
| CYP2E1 | 1571 | 10 | 135340867 | 135352620 |  |  |  |  |  |  | + |  |
| CYP2R1 | 120227 | 11 | 14899555 | 14913751 |  |  |  |  |  |  | + |  |
| CYP39A1 | 51302 | 6 | 46517445 | 46620523 |  |  |  |  |  |  | + |  |
| CYP3A4 | 1576 | 7 | 99354604 | 99381808 |  |  |  |  |  |  | + |  |
| CYP46A1 | 10858 | 14 | 100150755 | 100193638 |  |  |  |  |  |  | + |  |
| CYP4A11 | 1579 | 1 | 47394846 | 47407156 |  |  |  |  |  |  | + |  |
| CYP4B1 | 1580 | 1 | 47264670 | 47285021 |  |  |  |  |  |  | + |  |
| CYP51A1 | 1595 | 7 | 91741463 | 91764059 |  |  |  |  |  |  | + |  |
| CYP7A1 | 1581 | 8 | 59402737 | 59412720 |  |  |  |  |  |  | + |  |
| CYP7B1 | 9420 | 8 | 65508529 | 65711348 |  |  |  |  |  |  | + |  |
| CYP8B1 | 1582 | 3 | 42913684 | 42917633 |  |  |  |  |  |  | + |  |
| DAGLB | 221955 | 7 | 6409116 | 6448012 |  |  |  |  |  | + |  |  |
| ECI1 | 1632 | 16 | 2289873 | 2301602 |  |  |  |  |  |  | + |  |
| DECR1 | 1666 | 8 | 91013580 | 91064227 |  |  |  |  |  |  | + |  |
| DEGS1 | 8560 | 1 | 224370928 | 224381143 |  |  |  |  |  |  | + |  |
| DEGS2 | 123099 | 14 | 100612753 | 100626012 |  |  |  |  |  |  | + |  |
| DGAT1 | 8694 | 8 | 145538247 | 145550567 |  |  |  |  |  |  | + |  |
| DGAT2 | 84649 | 11 | 75479778 | 75512579 |  |  |  |  |  |  | + |  |
| DHCR24 | 1718 | 1 | 55315300 | 55352921 |  |  |  |  |  |  | + |  |
| DHCR7 | 1717 | 11 | 71145457 | 71159477 |  |  |  |  |  |  | + |  |
| DLG4 | 1742 | 17 | 7189890 | 7220050 |  |  |  |  |  | + |  |  |
| DNAH11 | 8701 | 7 | 21582833 | 21941457 |  |  | + |  |  |  |  |  |
| DOCK7 | 85440 | 1 | 62920397 | 63153969 | + |  |  |  |  |  |  |  |
| DPEP2 | 64174 | 16 | 68021293 | 68033364 |  |  |  |  |  |  | + |  |
| GLB1 | 2720 | 3 | 33038100 | 33138694 |  |  |  |  |  |  | + |  |
| ECHS1 | 1892 | 10 | 135175987 | 135186908 |  |  |  |  |  |  | + |  |
| EHBP1 | 23301 | 2 | 62673851 | 63046487 |  |  |  |  |  | + |  |  |
| ELOVL1 | 64834 | 1 | 43829072 | 43833699 |  |  |  |  |  |  | + |  |
| ELOVL2 | 54898 | 6 | 10980992 | 11044624 |  |  |  |  |  |  | + |  |
| ELOVL3 | 83401 | 10 | 103986143 | 103989346 |  |  |  |  |  |  | + |  |
| ELOVL4 | 6785 | 6 | 80624529 | 80657315 |  |  |  |  |  |  | + |  |
| ELOVL5 | 60481 | 6 | 53132196 | 53213942 |  |  |  |  |  |  | + |  |
| ELOVL6 | 79071 | 4 | 110970229 | 111119820 |  |  |  |  |  |  | + |  |
| ELOVL7 | 79993 | 5 | 60047616 | 60140101 |  |  |  |  |  |  | + |  |
| ERGIC3 | 51614 | 20 | 34129778 | 34145405 |  |  | + |  |  |  |  |  |
| EVI5 | 7813 | 1 | 92974253 | 93257961 |  |  | + |  |  |  |  |  |
| FABP1 | 2168 | 2 | 88422510 | 88427578 |  |  |  |  |  |  | + |  |
| FABP2 | 2169 | 4 | 120238405 | 120243316 |  |  |  |  |  |  | + |  |
| FABP4 | 2167 | 8 | 82390732 | 82395473 |  |  |  |  |  |  | + |  |
| FABP6 | 2172 | 5 | 159614374 | 159665729 |  |  |  |  |  |  | + |  |
| FADS1 | 3992 | 11 | 61567097 | 61584529 |  |  |  |  |  |  | + |  |
| FADS2 | 9415 | 11 | 61595713 | 61634825 |  |  | + |  |  |  |  |  |
| FADS3 | 3995 | 11 | 61640998 | 61659006 |  |  | + |  |  |  |  |  |
| FAM117B | 150864 | 2 | 202635178 | 202769757 |  |  |  |  |  | + |  |  |
| FAM13A | 10144 | 4 | 88725954 | 89111398 |  |  |  |  |  | + |  |  |
| FAR1 | 84188 | 11 | 13690206 | 13753893 |  |  |  |  |  |  | + |  |
| FAR2 | 55711 | 12 | 29376598 | 29487006 |  |  |  |  |  |  | + |  |
| FASN | 2194 | 17 | 80036214 | 80056106 |  |  |  |  |  |  | + |  |
| FDFT1 | 2222 | 8 | 11660190 | 11696818 |  |  |  |  |  |  | + |  |
| FDPS | 2224 | 1 | 155278539 | 155290457 |  |  |  |  |  |  | + |  |
| FFAR4 | 338557 | 10 | 93566204 | 93590072 |  |  |  |  |  |  |  | + |
| FGF21 | 26291 | 19 | 49259344 | 49261582 |  |  |  | + |  |  |  |  |
| FHL2 | 2274 | 2 | 105977283 | 106055230 |  |  |  |  |  |  | + |  |
| FN1 | 2335 | 2 | 215360440 | 215436167 |  |  |  |  |  | + |  |  |
| FOXC2 | 2303 | 16 | 86567251 | 86568933 |  |  |  |  | + |  |  |  |
| FRK | 2444 | 6 | 116262693 | 116381921 |  |  | + |  |  |  |  |  |
| FRMD5 | 84978 | 15 | 44165730 | 44487429 |  |  | + |  |  |  |  |  |
| FTO | 79068 | 16 | 53703963 | 54114467 |  |  |  |  |  | + |  |  |
| G0S2 | 50486 | 1 | 209675325 | 209676390 |  |  |  |  |  |  |  | + |
| GAL | 51083 | 11 | 68684515 | 68691175 |  |  |  |  | + |  |  |  |
| GALNT2 | 2590 | 1 | 230202956 | 230417875 | + | + |  |  |  |  |  |  |
| GC | 2638 | 4 | 72607410 | 72649888 |  |  |  |  |  |  | + |  |
| GCKR | 2646 | 2 | 27719706 | 27746551 | + | + |  |  |  |  |  |  |
| GGPS1 | 9453 | 1 | 235491753 | 235507847 |  |  |  |  |  |  | + |  |
| GGT5 | 2687 | 22 | 24615622 | 24641110 |  |  |  |  |  |  | + |  |
| GK | 2710 | X | 30671476 | 30748725 |  |  |  |  |  |  | + |  |
| GLIPR1 | 11010 | 12 | 75874513 | 75895716 |  |  |  |  |  |  | + |  |
| GNB1 | 2782 | 1 | 1716729 | 1822495 |  |  |  | + |  |  |  |  |
| GNPAT | 8443 | 1 | 231376919 | 231413719 |  |  |  |  |  |  | + |  |
| GOT2 | 2806 | 16 | 58741035 | 58768246 |  |  |  |  |  |  | + |  |
| GPAM | 57678 | 10 | 113909622 | 113943525 |  |  |  |  |  |  | + |  |
| GPAT2 | 150763 | 2 | 96687694 | 96700727 |  |  |  |  |  |  | + |  |
| GPD1 | 2819 | 12 | 50497801 | 50505096 |  |  |  |  |  |  | + |  |
| GPD2 | 2820 | 2 | 157291965 | 157442915 |  |  |  |  |  |  | + |  |
| GPIHBP1 | 338328 | 8 | 144295068 | 144299044 |  |  |  | + |  |  |  |  |
| HCAR2 | 338442 | 12 | 123185840 | 123187904 |  |  |  | + |  |  |  |  |
| HCAR3 | 8843 | 12 | 123199303 | 123201439 |  |  |  | + |  |  |  |  |
| GPR146 | 115330 | 7 | 1044573 | 1059269 |  |  |  |  |  | + |  |  |
| GRHL1 | 29841 | 2 | 10091792 | 10142412 |  |  |  |  |  |  | + |  |
| GRIN3A | 116443 | 9 | 104331634 | 104500862 |  | + |  |  |  |  |  |  |
| GSK3B | 2932 | 3 | 119821321 | 120094417 |  |  |  |  |  | + |  |  |
| HACL1 | 26061 | 3 | 15602239 | 15643130 |  |  |  |  |  |  | + |  |
| HADH | 3033 | 4 | 108910870 | 108956331 |  |  |  |  |  |  | + |  |
| HADHA | 3030 | 2 | 26413504 | 26467594 |  |  |  |  |  |  | + |  |
| HADHB | 3032 | 2 | 26467616 | 26513333 |  |  |  |  |  |  | + |  |
| HAS1 | 3036 | 19 | 51713112 | 51723992 |  |  |  |  |  | + |  |  |
| HBS1L | 10767 | 6 | 134960378 | 135054898 |  |  |  |  |  | + |  |  |
| HDAC3 | 8841 | 5 | 141000443 | 141016423 |  |  |  |  |  |  | + |  |
| HDGF | 3068 | 1 | 156742107 | 156752448 |  |  |  |  |  | + |  |  |
| HDLBP | 3069 | 2 | 242166679 | 242255254 |  |  |  | + |  |  |  |  |
| HFE | 3077 | 6 | 26087509 | 26095469 |  |  | + |  |  |  |  |  |
| HMGCL | 3155 | 1 | 24128367 | 24151949 |  |  |  |  |  |  | + |  |
| HMGCR | 3156 | 5 | 74632993 | 74657926 | + |  |  |  |  |  | + |  |
| HMGCS1 | 3157 | 5 | 43289493 | 43313595 |  |  |  |  |  |  | + |  |
| HMGCS2 | 3158 | 1 | 120290619 | 120311555 |  |  |  |  |  |  | + |  |
| HNF1A | 6927 | 12 | 121416549 | 121440315 |  |  | + |  |  |  |  |  |
| HNF4A | 3172 | 20 | 42984441 | 43061485 |  |  | + |  |  |  |  |  |
| HPR | 3250 | 16 | 72097125 | 72111145 |  |  | + |  |  |  |  |  |
| HSD11B1 | 3290 | 1 | 209859550 | 209908295 |  |  |  |  |  |  | + |  |
| HSD17B1 | 3292 | 17 | 40703984 | 40707232 |  |  |  |  |  |  | + |  |
| HSD17B12 | 51144 | 11 | 43702143 | 43878169 |  |  |  |  |  |  | + |  |
| HSD17B3 | 3293 | 9 | 98997589 | 99064434 |  |  |  |  |  |  | + |  |
| HSD17B4 | 3295 | 5 | 118788148 | 118878027 |  |  |  |  |  |  | + |  |
| HSD17B7 | 51478 | 1 | 162760496 | 162782608 |  |  |  |  |  |  | + |  |
| HSD3B1 | 3283 | 1 | 120049826 | 120057681 |  |  |  |  |  |  | + |  |
| HSD3B2 | 3284 | 1 | 119957554 | 119965662 |  |  |  |  |  |  | + |  |
| HSD3B7 | 80270 | 16 | 30996519 | 31000473 |  |  |  |  |  |  | + |  |
| HSPG2 | 3339 | 1 | 22148737 | 22222804 |  |  |  |  |  |  | + |  |
| IDH1 | 3417 | 2 | 209100953 | 209119806 |  |  |  |  |  |  | + |  |
| IDI1 | 3422 | 10 | 1085963 | 1095061 |  |  |  |  |  |  | + |  |
| IDI2 | 91734 | 10 | 1064847 | 1071799 |  |  |  |  |  |  | + |  |
| IKZF1 | 10320 | 7 | 50303453 | 50405101 |  |  |  |  |  | + |  |  |
| INSIG1 | 3638 | 7 | 155089486 | 155101945 |  |  |  |  |  |  |  | + |
| INSIG2 | 51141 | 2 | 118846050 | 118867597 |  |  |  |  |  |  |  | + |
| INSR | 3643 | 19 | 7112255 | 7294405, |  |  |  |  |  | + |  |  |
| IRF2BP2 | 359948 | 1 | 234740015 | 234745271 |  |  | + |  |  |  |  |  |
| IRS1 | 3667 | 2 | 227596033 | 227663506 |  |  | + |  |  |  |  |  |
| JMJD1C | 221037 | 10 | 64926985 | 65225722 |  |  | + |  |  |  |  |  |
| JMJD7-PLA2G4B | 8681 | 15 | 42120283 | 42140346 |  |  |  |  |  |  | + |  |
| KAT5 | 10524 | 11 | 65711996 | 65719606 |  |  |  |  |  | + |  |  |
| KCNK17 | 89822 | 6 | 39299001 | 39314461 |  |  |  |  |  | + |  |  |
| KDSR | 2531 | 18 | 60994971 | 61034506 |  |  |  |  |  |  | + |  |
| KLF13 | 51621 | 15 | 31619083 | 31670102 |  |  |  | + |  |  |  |  |
| KLF14 | 136259 | 7 | 130417396 | 130418888 |  |  |  | + |  |  |  |  |
| KLHL12 | 59349 | 1 | 202860230 | 202896371 |  |  |  |  |  |  |  | + |
| KLHL8 | 57563 | 4 | 88082214 | 88141674 |  |  | + |  |  |  |  |  |
| LACTB | 114294 | 15 | 63413999 | 63434260 |  |  | + |  |  |  |  |  |
| CERS1 | 10715 | 19 | 18979361 | 19006953 |  |  |  |  |  |  | + |  |
| CERS3 | 204219 | 15 | 100940600 | 101084925 |  |  |  |  |  |  | + |  |
| CERS5 | 91012 | 12 | 50523581 | 50561097 |  |  |  |  |  |  | + |  |
| CERS6 | 253782 | 2 | 169312835 | 169631152 |  |  |  |  |  |  | + |  |
| LBR | 3930 | 1 | 225589204 | 225616519 |  |  |  |  |  |  | + |  |
| LCAT | 3931 | 16 | 67973787 | 67978015 |  | + |  |  |  |  | + |  |
| LDLR | 3949 | 19 | 11200057 | 11244506 | + | + |  |  |  |  | + |  |
| LDLRAP1 | 26119 | 1 | 25870076 | 25895377 |  |  |  |  |  |  | + |  |
| LEPR | 3953 | 1 | 65420652 | 65637493 |  |  |  |  | + |  |  |  |
| LGMN | 5641 | 14 | 93170152 | 93215047 |  |  |  |  |  |  | + |  |
| LILRA3 | 11026 | 19 | 54799854 | 54804265 |  |  | + |  |  |  |  |  |
| LINC01101 | 84931 | 2 | 120464335 | 120466349 |  |  |  |  |  | + |  |  |
| LIPA | 3988 | 10 | 90973326 | 91011660 |  |  |  |  |  |  |  | + |
| LIPC | 3990 | 15 | 58724175 | 58861073 | + | + |  |  |  |  | + |  |
| LIPE | 3991 | 19 | 42905666 | 42931578 |  |  |  |  |  |  | + |  |
| LIPF | 8513 | 10 | 90424094 | 90438572 |  |  |  |  |  |  | + |  |
| LIPG | 9388 | 18 | 47088427 | 47119278 | + | + |  |  |  |  |  |  |
| LMF1 | 64788 | 16 | 903634 | 1031318 |  |  |  |  |  |  |  | + |
| LMF2 | 91289 | 22 | 50941376 | 50946135 |  |  |  |  |  |  |  | + |
| LPA | 4018 | 6 | 160952515 | 161087407 |  |  |  |  |  |  | + |  |
| LPCAT3 | 10162 | 12 | 7085347 | 7125842 |  |  |  |  |  |  |  | + |
| LPIN1 | 23175 | 2 | 11886740 | 11967535 |  |  |  |  |  |  | + |  |
| LPIN2 | 9663 | 18 | 2916992 | 3011945 |  |  |  |  |  |  | + |  |
| LPIN3 | 64900 | 20 | 39969560 | 39989222 |  |  |  |  |  |  | + |  |
| LPL | 4023 | 8 | 19796582 | 19824770 | + | + |  |  |  |  | + |  |
| LRAT | 9227 | 4 | 155665163 | 155674270 |  |  |  |  |  |  | + |  |
| LRP1 | 4035 | 12 | 57522282 | 57607142 |  |  |  |  |  |  | + |  |
| LRP2 | 4036 | 2 | 169983619 | 170219122 |  |  |  |  |  |  | + |  |
| LRP4 | 4038 | 11 | 46878268 | 46940173 |  |  | + |  |  | + |  |  |
| LRP8 | 7804 | 1 | 53711212 | 53793821 |  |  |  |  |  |  |  | + |
| LSS | 4047 | 21 | 47608360 | 47648738 |  |  |  |  |  |  | + |  |
| LTA4H | 4048 | 12 | 96394611 | 96429365 |  |  |  |  |  |  | + |  |
| LTC4S | 4056 | 5 | 179220986 | 179223513 |  |  |  |  |  |  | + |  |
| MAFB | 9935 | 20 | 39314515 | 39317876 |  |  | + |  |  |  |  |  |
| MAMSTR | 284358 | 19 | 49216255 | 49222976 |  |  | + |  |  |  |  |  |
| MAP3K1 | 4214 | 5 | 56110900 | 56191979 |  |  | + |  |  |  |  |  |
| MAPKAPK2 | 9261 | 1 | 206858289 | 206907626 |  |  |  |  |  |  | + |  |
| MARC1 | 64757 | 1 | 220960039 | 220987741 |  |  | + |  |  |  |  |  |
| MARCH8 | 220972 | 10 | 45454585 | 45594907 |  |  |  |  |  | + |  |  |
| MBTPS1 | 8720 | 16 | 84087368 | 84150517 |  |  |  |  |  |  |  | + |
| MBTPS2 | 51360 | X | 21857656 | 21903541 |  |  |  |  |  |  |  | + |
| MC4R | 4160 | 18 | 58038564 | 58040001 |  |  |  |  |  |  |  | + |
| MCEE | 84693 | 2 | 71336806 | 71357394 |  |  |  |  |  |  | + |  |
| ME1 | 4199 | 6 | 83920108 | 84140938 |  |  |  |  |  |  | + |  |
| MED1 | 5469 | 17 | 37560538 | 37607527 |  |  |  |  |  |  | + |  |
| MET | 4233 | 7 | 116672359 | 116798386 |  |  |  |  |  | + |  |  |
| MGLL | 11343 | 3 | 127407909 | 127542051 |  |  |  |  |  |  | + |  |
| MIR122 | 406906 | 18 | 56118306 | 56118390 |  |  |  |  |  |  |  | + |
| MIR148A | 406940 | 7 | 25949919 | 25949986 |  |  |  |  |  | + |  |  |
| MIR33A | 407039 | 22 | 42296948 | 42297016 |  |  |  |  |  |  |  | + |
| MIR370 | 442915 | 14 | 81431308 | 81431382 |  |  |  |  |  |  |  | + |
| MLXIPL | 51085 | 7 | 73007524 | 73038870 | + | + |  |  |  |  |  |  |
| MMAB | 326625 | 12 | 109991520 | 110011358 |  | + |  |  |  |  |  |  |
| MOGAT2 | 80168 | 11 | 75428934 | 75442331 |  |  |  |  |  |  | + |  |
| MOGAT3 | 346606 | 7 | 100839010 | 100844302 |  |  |  |  |  |  | + |  |
| MPP3 | 4356 | 17 | 43800799 | 43833192 |  |  |  |  |  | + |  |  |
| MSL2 | 55167 | 3 | 135867760 | 135914688 |  |  | + |  |  |  |  |  |
| MSR1 | 4481 | 8 | 15965387 | 16050300 |  |  |  |  |  |  |  | + |
| MTMR3 | 8897 | 22 | 29883165 | 30030868 |  |  |  |  |  | + |  |  |
| MTOR | 2475 | 1 | 11166588 | 11322608 |  |  | + |  |  |  |  |  |
| MTTP | 4547 | 4 | 100485240 | 100545154 |  |  |  |  |  |  | + |  |
| MUT | 4594 | 6 | 49398073 | 49431041 |  |  |  |  |  |  | + |  |
| MVD | 4597 | 16 | 88718348 | 88729495 |  |  |  |  |  |  | + |  |
| MVK | 4598 | 12 | 110011500 | 110035071 |  | + |  |  |  |  | + |  |
| MYBPHL | 343263 | 1 | 109834987 | 109849663 | + |  |  |  |  |  |  |  |
| MYL5 | 4636 | 4 | 671711 | 675817 |  |  |  | + |  |  |  |  |
| MYLIP | 29116 | 6 | 16129317 | 16148479 |  |  |  |  |  |  |  |  |
| NAT2 | 10 | 8 | 18248755 | 18258723 |  |  | + |  |  |  |  |  |
| NCAN | 1463 | 19 | 19322782 | 19363061 |  | + |  |  |  |  |  |  |
| NCOA1 | 8648 | 2 | 24807346 | 24993571 |  |  |  |  |  |  | + |  |
| NCOA2 | 10499 | 8 | 71024267 | 71316020 |  |  |  |  |  |  | + |  |
| NCOA3 | 8202 | 20 | 46130601 | 46285621 |  |  |  |  |  |  | + |  |
| NCOA6 | 23054 | 20 | 33302578 | 33413433 |  |  |  |  |  |  | + |  |
| NCOR1 | 9611 | 17 | 15933408 | 16118874 |  |  |  |  |  |  | + |  |
| NCOR2 | 9612 | 12 | 124808961 | 125052010 |  |  |  |  |  |  | + |  |
| NDST1 | 3340 | 5 | 150485823 | 150558211 |  |  |  |  |  |  |  | + |
| NINJ2 | 4815 | 12 | 673462 | 772755 |  |  |  | + |  |  |  |  |
| NPC1 | 4864 | 18 | 21111463 | 21166581 |  |  |  |  |  |  |  | + |
| NPC1L1 | 29881 | 7 | 44552134 | 44580914 |  |  |  |  |  |  | + |  |
| NR0B2 | 8431 | 1 | 26911484 | 26914076 |  |  |  |  |  | + |  |  |
| NR1H3 | 10062 | 11 | 47270449 | 47290401 |  |  |  |  |  |  | + |  |
| NR1H4 | 9971 | 12 | 100867679 | 100957643 |  |  |  |  |  |  | + |  |
| NR1I2 | 8856 | 3 | 119499331 | 119537332 |  |  |  |  |  |  | + |  |
| NR1I3 | 9970 | 1 | 161199456 | 161208000 |  |  |  |  |  |  | + |  |
| NR2F1 | 7025 | 5 | 92919043 | 92929788 |  |  |  |  |  |  |  | + |
| NR2F2 | 7026 | 15 | 96869157 | 96883492 |  |  |  |  |  |  |  | + |
| NR5A2 | 2494 | 1 | 200027602 | 200177424 |  |  |  |  |  |  |  | + |
| NSDHL | 50814 | X | 151999511 | 152037907 |  |  |  |  |  |  | + |  |
| NYNRIN | 57523 | 14 | 24867992 | 24888494 |  |  | + |  |  |  |  |  |
| OLR1 | 4973 | 12 | 10310899 | 10324790 |  |  |  |  |  |  |  | + |
| OR4C46 | 119749 | 11 | 54603069 | 54603998 |  |  |  |  |  | + |  |  |
| OSBP | 5007 | 11 | 59341871 | 59383617 |  |  |  |  |  |  | + |  |
| OSBPL10 | 114884 | 3 | 31660825 | 31981850 |  |  |  |  | + |  |  |  |
| OSBPL1A | 114876 | 18 | 21742009 | 21977790 |  |  |  |  |  |  |  | + |
| OSBPL3 | 26031 | 7 | 24796537 | 24980212 |  |  |  |  |  |  |  | + |
| OSBPL6 | 114880 | 2 | 179059208 | 179264160 |  |  |  |  |  |  |  | + |
| OSBPL7 | 114881 | 17 | 45884733 | 45899147 |  |  | + |  |  |  |  |  |
| OXCT1 | 5019 | 5 | 41730167 | 41870791 |  |  |  |  |  |  | + |  |
| P4HB | 5034 | 17 | 79801034 | 79818544 |  |  |  |  |  |  | + |  |
| PABPC4 | 8761 | 1 | 40026485 | 40042521 |  |  | + |  |  | + |  |  |
| PAFAH1B2 | 5049 | 11 | 117144284 | 117178173 |  |  |  |  |  | + |  |  |
| PBX4 | 80714 | 19 | 19672522 | 19729439 | + |  |  |  |  |  |  |  |
| PCCA | 5095 | 13 | 100741269 | 101182691 |  |  |  |  |  |  | + |  |
| PCCB | 5096 | 3 | 135969167 | 136049013 |  |  |  |  |  |  | + |  |
| PCDH15 | 65217 | 10 | 53802771 | 54801291 |  |  |  |  | + |  |  |  |
| PCOLCE2 | 26577 | 3 | 142536702 | 142608045 |  |  |  |  |  |  |  | + |
| PCSK7 | 9159 | 11 | 117075788 | 117102811 |  |  |  |  |  |  |  | + |
| PCSK9 | 255738 | 1 | 55505149 | 55530526 | + | + |  |  |  |  |  |  |
| PCTP | 58488 | 17 | 53828356 | 53854748 |  |  |  |  |  |  |  | + |
| PCYT1A | 5130 | 3 | 195965253 | 196014584 |  |  |  |  |  |  |  | + |
| PCYT1B | 9468 | X | 24576204 | 24690979 |  |  |  |  |  |  |  | + |
| PDE3A | 5139 | 12 | 20522179 | 20837041 |  |  | + |  |  |  |  |  |
| PDIA2 | 64714 | 16 | 333118 | 337209 |  |  |  |  |  |  | + |  |
| PDXDC1 | 23042 | 16 | 14974591 | 15139410 |  |  |  |  |  | + |  |  |
| PDZK1 | 5174 | 1 | 145727666 | 145764207 |  |  |  |  |  |  |  | + |
| PEMT | 10400 | 17 | 17408877 | 17494994 |  |  |  |  |  |  |  | + |
| PEPD | 5184 | 19 | 33386949 | 33521893 |  |  |  |  |  | + |  |  |
| PEX11A | 8800 | 15 | 90226284 | 90233958 |  |  |  |  |  |  | + |  |
| PGLYRP2 | 114770 | 19 | 15579456 | 15590315 |  |  |  |  |  |  |  | + |
| PGS1 | 9489 | 17 | 76374735 | 76420640 |  |  | + |  |  |  |  |  |
| PHC1 | 1911 | 12 | 8914664 | 8941467 |  |  |  |  |  | + |  |  |
| PHLDB1 | 23187 | 11 | 118606296 | 118658038 |  |  |  |  |  | + |  |  |
| PHYH | 5264 | 10 | 13319796 | 13342130 |  |  |  |  |  |  | + |  |
| PIGC | 5279 | 1 | 172441457 | 172444090 |  |  |  |  |  | + |  |  |
| PINX1 | 54984 | 8 | 10622884 | 10697299 |  |  | + |  |  |  |  |  |
| PKD1L3 | 342372 | 16 | 71929538 | 71999978 |  |  |  |  |  |  |  | + |
| PLA2G10 | 8399 | 16 | 14766405 | 14788526 |  |  |  |  |  |  | + |  |
| PLA2G12A | 81579 | 4 | 110631145 | 110651242 |  |  |  |  |  |  | + |  |
| PLA2G12B | 84647 | 10 | 74694938 | 74714510 |  |  |  |  |  |  | + |  |
| PLA2G1B | 5319 | 12 | 120759914 | 120765592 |  |  |  |  |  |  | + |  |
| PLA2G2A | 5320 | 1 | 20301924 | 20306932 |  |  |  |  |  |  | + |  |
| PLA2G2C | 391013 | 1 | 20490484 | 20501687 |  |  |  |  |  |  | + |  |
| PLA2G2D | 26279 | 1 | 20438432 | 20446008 |  |  |  |  |  |  | + |  |
| PLA2G2E | 30814 | 1 | 20246800 | 20250110 |  |  |  |  |  |  | + |  |
| PLA2G2F | 64600 | 1 | 20465823 | 20476879 |  |  |  |  |  |  | + |  |
| PLA2G3 | 50487 | 22 | 31530793 | 31536469 |  |  |  |  |  |  | + |  |
| PLA2G4A | 5321 | 1 | 186798032 | 186958113 |  |  |  |  |  |  | + |  |
| PLA2G4B | 100137049 | 15 | 42131011 | 42140346 |  |  |  |  |  |  | + |  |
| PLA2G4E | 123745 | 15 | 42275952 | 42302445 |  |  |  |  |  |  | + |  |
| PLA2G5 | 5322 | 1 | 20396701 | 20418394 |  |  |  |  |  |  | + |  |
| PLA2G6 | 8398 | 22 | 38507502 | 38577761 |  |  |  |  |  |  | + |  |
| PLB1 | 151056 | 2 | 28718938 | 28866654 |  |  |  |  |  |  | + |  |
| PLD2 | 5338 | 17 | 4807101 | 4823432 |  |  |  |  |  | + |  |  |
| PLEC | 5339 | 8 | 144989321 | 145050913 |  |  | + |  |  |  |  |  |
| PLIN1 | 5346 | 15 | 90207598 | 90222648 |  |  |  |  |  |  | + |  |
| PLIN2 | 123 | 9 | 19115759 | 19127573 |  |  |  |  |  |  | + |  |
| PLTP | 5360 | 20 | 44527397 | 44540786 |  |  |  |  |  |  | + |  |
| PMVK | 10654 | 1 | 154897208 | 154909484 |  |  |  |  |  |  | + |  |
| PNLIP | 5406 | 10 | 118305428 | 118327367 |  |  |  |  |  |  | + |  |
| PNLIPRP1 | 5407 | 10 | 118350490 | 118368686 |  |  |  |  |  |  | + |  |
| PNLIPRP2 | 5408 | 10 | 118380465 | 118404654 |  |  |  |  |  |  | + |  |
| PNPLA2 | 57104 | 11 | 818901 | 825573 |  |  |  |  |  |  |  | + |
| PNPLA3 | 80339 | 22 | 43923739 | 43947568 |  |  |  |  |  |  |  | + |
| PNPLA5 | 150379 | 22 | 44275558 | 44287893 |  |  |  |  |  |  |  | + |
| PON1 | 5444 | 7 | 95298357 | 95324572 |  |  |  |  | + |  |  |  |
| PON3 | 5446 | 7 | 94989181 | 95025702 |  |  |  |  | + |  |  |  |
| PPAP2A | 8611 | 5 | 54720682 | 54830873 |  |  |  |  |  |  | + |  |
| PPAP2B | 8613 | 1 | 56960433 | 57045257 |  |  |  |  |  |  | + |  |
| PPAP2C | 8612 | 19 | 281043 | 291435 |  |  |  |  |  |  | + |  |
| PPARA | 5465 | 22 | 46546499 | 46639653 |  |  |  |  |  |  | + |  |
| PPARD | 5467 | 6 | 35310335 | 35395968 |  |  |  |  |  |  | + |  |
| PPARG | 5468 | 3 | 12329349 | 12475855 |  |  |  |  |  |  | + |  |
| PPARGC1A | 10891 | 4 | 23793644 | 23891700 |  |  |  |  |  |  |  | + |
| PPARGC1B | 133522 | 5 | 149109815 | 149234585 |  |  |  |  |  |  |  | + |
| PPM1L | 151742 | 3 | 160473996 | 160788817 |  |  |  |  |  |  | + |  |
| PPP1CA | 5499 | 11 | 67165652 | 67169376 |  |  |  |  |  |  | + |  |
| PPP1CB | 5500 | 2 | 28974614 | 29025806 |  |  |  |  |  |  | + |  |
| PPP1CC | 5501 | 12 | 111157615 | 111180757 |  |  |  |  |  |  | + |  |
| PPP1R3B | 79660 | 8 | 8993764 | 9009152 |  |  | + |  |  |  |  |  |
| PRIC285 | 85441 | 20 | 62189439 | 62205592 |  |  |  |  |  |  | + |  |
| PRKAA2 | 5563 | 1 | 57110990 | 57181008 |  |  |  |  |  |  | + |  |
| PRKAB2 | 5565 | 1 | 146626685 | 146644129 |  |  |  |  |  |  | + |  |
| PRKACA | 5566 | 19 | 14202500 | 14228559 |  |  |  |  |  |  | + |  |
| PRKACB | 5567 | 1 | 84543745 | 84704181 |  |  |  |  |  |  | + |  |
| PRKACG | 5568 | 9 | 71627445 | 71629039 |  |  |  |  |  |  | + |  |
| PRKAG2 | 51422 | 7 | 151253200 | 151574316 |  |  |  |  |  |  | + |  |
| PRKCA | 5578 | 17 | 66302640 | 66810744 |  |  |  |  |  | + |  |  |
| PRKD1 | 5587 | 14 | 30045685 | 30396899 |  |  |  |  |  |  | + |  |
| PRMT2 | 3275 | 21 | 48055527 | 48084863 |  |  |  | + |  |  |  |  |
| PSRC1 | 84722 | 1 | 109822176 | 109825790 | + | + |  |  |  |  |  |  |
| PTGES3 | 10728 | 12 | 57057125 | 57082078 |  |  |  |  |  |  | + |  |
| PTGIS | 5740 | 20 | 48120411 | 48184707 |  |  |  |  |  |  | + |  |
| PTGS1 | 5742 | 9 | 125133229 | 125157981 |  |  |  |  |  |  | + |  |
| PTGS2 | 5743 | 1 | 186640944 | 186649559 |  |  |  |  |  |  | + |  |
| PXN | 5829 | 12 | 120210439 | 120265771 |  |  |  |  |  | + |  |  |
| RAB3GAP1 | 22930 | 2 | 135809835 | 135928280 |  |  | + |  |  |  |  |  |
| RAF1 | 5894 | 3 | 12625100 | 12705700 |  |  | + |  |  |  |  |  |
| RARA | 5914 | 17 | 38465423 | 38513895 |  |  |  |  |  |  | + |  |
| RARB | 5915 | 3 | 25469754 | 25639423 |  |  |  |  |  |  | + |  |
| RARG | 5916 | 12 | 53604353 | 53626036 |  |  |  |  |  |  | + |  |
| RBKS | 64080 | 2 | 28004266 | 28113223 |  | + |  |  |  |  |  |  |
| RBM5 | 10181 | 3 | 50088908 | 50118964 |  |  |  |  |  | + |  |  |
| RBP2 | 5948 | 3 | 139171726 | 139195352 |  |  |  |  |  |  | + |  |
| RDH11 | 51109 | 14 | 68143517 | 68162510 |  |  |  |  |  |  | + |  |
| RGL1 | 23179 | 1 | 183605208 | 183897666 |  |  |  |  |  |  | + |  |
| RHOA | 387 | 3 | 49396578 | 49449526 |  |  |  |  |  |  |  | + |
| RORA | 6095 | 15 | 60780483 | 61521502 |  |  |  |  |  |  |  | + |
| RORB | 6096 | 9 | 77112252 | 77302117 |  |  |  |  |  |  |  | + |
| RORC | 6097 | 1 | 151778547 | 151804348 |  |  |  |  |  |  |  | + |
| RSPO3 | 84870 | 6 | 127118671 | 127199481 |  |  |  |  |  | + |  |  |
| RXRA | 6256 | 9 | 137218316 | 137332431 |  |  |  |  |  |  | + |  |
| RXRG | 6258 | 1 | 165370159 | 165414592 |  |  |  |  |  |  |  | + |
| SAR1B | 51128 | 5 | 133936839 | 133968533 |  |  |  |  |  |  | + |  |
| SBNO1 | 55206 | 12 | 123773656 | 123834988 |  |  | + |  |  |  |  |  |
| MSMO1 | 6307 | 4 | 166248818 | 166264225 |  |  |  |  |  |  | + |  |
| SC5D | 6309 | 11 | 121163388 | 121184119 |  |  |  |  |  |  | + |  |
| SCAP | 22937 | 3 | 47455184 | 47517445 |  |  |  |  |  |  |  | + |
| SCARB1 | 949 | 12 | 125262174 | 125348519 |  |  |  |  |  |  | + |  |
| SCP2 | 6342 | 1 | 53392901 | 53517289 |  |  |  |  |  |  | + |  |
| SDC1 | 6382 | 2 | 20400558 | 20425194 |  |  |  |  |  |  | + |  |
| SETD2 | 29072 | 3 | 47016408 | 47164109 |  |  |  |  |  | + |  |  |
| SETDB2 | 83852 | 13 | 49444293 | 49495003 |  |  |  |  |  |  |  | + |
| SGMS1 | 259230 | 10 | 52065345 | 52383737 |  |  |  |  |  |  | + |  |
| SGMS2 | 166929 | 4 | 108745721 | 108836203 |  |  |  |  |  |  | + |  |
| SGPL1 | 8879 | 10 | 72575704 | 72640946 |  |  |  |  |  |  | + |  |
| SGPP1 | 81537 | 14 | 64150934 | 64194756 |  |  |  |  |  |  | + |  |
| SGPP2 | 130367 | 2 | 223289322 | 223423617 |  |  |  |  |  |  | + |  |
| SIN3A | 25942 | 15 | 75661720 | 75748124 |  |  |  |  |  |  | + |  |
| SIN3B | 23309 | 19 | 16940218 | 16991164 |  |  |  |  |  |  | + |  |
| SLC10A1 | 6554 | 14 | 70242552 | 70264006 |  |  |  |  |  |  | + |  |
| SLC10A2 | 6555 | 13 | 103696348 | 103719196 |  |  |  |  |  |  | + |  |
| SLC22A5 | 6584 | 5 | 132369704 | 132395614 |  |  |  |  |  |  |  | + |
| SLC25A1 | 6576 | 22 | 19163094 | 19166301 |  |  |  |  |  |  | + |  |
| SLC25A17 | 10478 | 22 | 41165636 | 41215392 |  |  |  |  |  |  | + |  |
| SLC25A20 | 788 | 3 | 48894356 | 48936402 |  |  |  |  |  |  | + |  |
| SLC25A40 | 55972 | 7 | 87834499 | 87876377 |  |  |  |  |  | + |  |  |
| SLC27A2 | 11001 | 15 | 50474393 | 50528589 |  |  |  |  |  |  | + |  |
| SLC27A4 | 10999 | 9 | 131102839 | 131123749 |  |  |  |  |  |  | + |  |
| SLC27A5 | 10998 | 19 | 59009700 | 59023432 |  |  |  |  |  |  | + |  |
| SLC2A2 | 6514 | 3 | 170714137 | 170744768 |  |  |  |  |  |  | + |  |
| SLC2A4 | 6517 | 17 | 7281735 | 7288048 |  |  |  |  |  |  |  | + |
| SLC39A8 | 64116 | 4 | 103172198 | 103266655 |  |  | + |  |  | + |  |  |
| SLC7A10 | 56301 | 19 | 33699570 | 33716756 |  |  |  | + |  |  |  |  |
| SLCO1A2 | 6579 | 12 | 21417534 | 21548371 |  |  |  |  |  |  | + |  |
| SLCO1B1 | 10599 | 12 | 21284128 | 21392730 |  |  |  |  |  |  | + |  |
| SLCO1B3 | 28234 | 12 | 20963638 | 21069658 |  |  |  |  |  |  | + |  |
| SMARCD3 | 6604 | 7 | 150936059 | 150974231 |  |  |  |  |  |  | + |  |
| SMIM20 | 389203 | 4 | 25914192 | 25929879 |  |  |  |  |  | + |  |  |
| SNX13 | 23161 | 7 | 17790761 | 17940518 |  |  |  |  |  | + |  |  |
| SNX5 | 27131 | 20 | 17941596 | 17968991 |  |  |  |  |  | + |  |  |
| SOAT1 | 6646 | 1 | 179263017 | 179324453 |  |  |  |  |  |  | + |  |
| SORT1 | 6272 | 1 | 109852192 | 109940563 | + | + |  |  |  |  |  |  |
| SOX17 | 64321 | 8 | 54457935 | 54460896 |  |  |  |  |  | + |  |  |
| SPHK1 | 8877 | 17 | 74380690 | 74383941 |  |  |  |  |  |  | + |  |
| SPHK2 | 56848 | 19 | 49122548 | 49133662 |  |  |  |  |  |  | + |  |
| SPTLC1 | 10558 | 9 | 94793427 | 94877690 |  |  |  |  |  |  | + |  |
| SPTLC2 | 9517 | 14 | 77973270 | 78083110 |  |  |  |  |  |  | + |  |
| SPTLC3 | 55304 | 20 | 12989627 | 13147411 |  |  |  |  |  |  | + |  |
| SPTY2D1 | 144108 | 11 | 18627948 | 18656020 |  |  | + |  |  |  |  |  |
| SQLE | 6713 | 8 | 126010720 | 126034525 |  |  |  |  |  |  | + |  |
| SRD5A1 | 6715 | 5 | 6633500 | 6669675 |  |  |  |  |  |  | + |  |
| SRD5A2 | 6716 | 2 | 31749656 | 31806040 |  |  |  |  |  |  | + |  |
| SRD5A3 | 79644 | 4 | 56212409 | 56237866 |  |  |  |  |  |  | + |  |
| SREBF1 | 6720 | 17 | 17714663 | 17740325 |  |  |  |  |  |  |  | + |
| SREBF2 | 6721 | 22 | 42229106 | 42302375 |  |  |  |  |  |  |  | + |
| ST3GAL4 | 6484 | 11 | 126225540 | 126284536 |  |  | + |  |  |  |  |  |
| STAB1 | 23166 | 3 | 52495338 | 52524496 |  |  |  |  |  | + |  |  |
| STAP1 | 26228 | 4 | 68424446 | 68473055 |  |  |  |  |  |  |  |  |
| STAR | 6770 | 8 | 38000218 | 38008600 |  |  |  |  |  |  | + |  |
| STARD3 | 10948 | 17 | 37793333 | 37820454 |  |  |  |  |  |  |  | + |
| STARD4 | 134429 | 5 | 110834022 | 110848157 |  |  |  |  |  |  | + |  |
| STARD5 | 80765 | 15 | 81605007 | 81616524 |  |  |  |  |  |  | + |  |
| STARD6 | 147323 | 18 | 51851062 | 51880943 |  |  |  |  |  |  | + |  |
| SUGP1 | 57794 | 19 | 19387322 | 19431307 |  | + |  |  |  |  |  |  |
| SULT2A1 | 6822 | 19 | 48373723 | 48389654 |  |  |  |  |  |  | + |  |
| TBL1X | 6907 | X | 9431335 | 9687780 |  |  |  |  |  |  | + |  |
| TBL1XR1 | 79718 | 3 | 176738542 | 176915048 |  |  |  |  |  |  | + |  |
| TBL2 | 26608 | 7 | 72983274 | 72993013 | + |  |  |  |  |  |  |  |
| TBXAS1 | 6916 | 7 | 139478047 | 139720125 |  |  |  |  |  |  | + |  |
| TCF7L2 | 6934 | 10 | 112950219 | 113167678 |  |  |  |  | + |  |  |  |
| TEAD2 | 8463 | 19 | 49340595 | 49362457 |  |  |  |  |  |  |  | + |
| TECR | 9524 | 19 | 14640382 | 14676792 |  |  |  |  |  |  | + |  |
| TGS1 | 96764 | 8 | 56685791 | 56738005 |  |  |  |  |  |  | + |  |
| TIAM2 | 26230 | 6 | 155411423 | 155578857 |  |  |  |  |  |  | + |  |
| TIMD4 | 91937 | 5 | 156346293 | 156390266 |  |  | + |  |  |  |  |  |
| TM6SF2 | 53345 | 19 | 19264365 | 19273265 |  |  |  |  |  |  |  | + |
| TM7SF2 | 7108 | 11 | 64879341 | 64883707 |  |  |  |  |  |  | + |  |
| TMEM176A | 55365 | 7 | 150800543 | 150805120 |  |  |  |  |  | + |  |  |
| TMEM97 | 27346 | 17 | 26646121 | 26655711 |  |  |  |  |  |  |  | + |
| TNFRSF1B | 7133 | 1 | 12167003 | 12209220) |  |  |  |  | + |  |  |  |
| TNFRSF21 | 27242 | 6 | 47199268 | 47277680 |  |  |  |  |  |  | + |  |
| TOM1 | 10043 | 22 | 35299275 | 35347994 |  |  |  |  |  | + |  |  |
| TOP1 | 7150 | 20 | 39657462 | 39753127 |  |  | + |  |  |  |  |  |
| TPMT | 7172 | 6 | 18128542 | 18155374 |  |  |  | + |  |  |  |  |
| TRIB1 | 10221 | 8 | 126442563 | 126450645 | + | + |  |  |  |  |  |  |
| TRIB3 | 57761 | 20 | 361308 | 378203 |  |  |  |  |  |  | + |  |
| TRPS1 | 7227 | 8 | 116420724 | 116681228 |  |  | + |  |  | + |  |  |
| TTC39B | 158219 | 9 | 15170842 | 15307358 |  |  | + |  |  |  |  |  |
| TXNRD1 | 7296 | 12 | 104609559 | 104744062 |  |  |  |  |  |  | + |  |
| TYW1B | 441250 | 7 | 72023729 | 72298813 |  |  | + |  |  |  |  |  |
| UBASH3B | 84959 | 11 | 122526398 | 122685187 |  |  | + |  |  |  |  |  |
| UBE2L3 | 7332 | 22 | 21903736 | 21978323 |  |  | + |  |  |  |  |  |
| UCP1 | 7350 | 4 | 141481050 | 141489959 |  |  |  |  |  |  | + |  |
| UGT1A1 | 54658 | 2 | 233760273 | 233773299 |  |  |  |  |  | + |  |  |
| UGT1A9 | 54600 | 2 | 234580544 | 234681951 |  |  |  |  |  |  | + |  |
| USF1 | 7391 | 1 | 161039251 | 161045979 |  |  |  |  | + |  |  |  |
| USP3 | 9960 | 15 | 63796810 | 63883663 |  |  |  |  |  |  |  | + |
| VAPA | 9218 | 18 | 9913955 | 9960018 |  |  |  |  |  |  | + |  |
| VAPB | 9217 | 20 | 56964245 | 57021963 |  |  |  |  |  |  | + |  |
| VDR | 7421 | 12 | 48235320 | 48298814 |  |  |  |  |  |  | + |  |
| VEGFA | 7422 | 6 | 43770209 | 43786487 |  |  |  |  |  | + |  |  |
| VIM | 7431 | 10 | 17227935 | 17237593 |  |  |  |  |  | + |  |  |
| VLDLR | 7436 | 9 | 2621793 | 2654485 |  |  |  |  |  |  |  | + |
| WWOX | 51741 | 16 | 78133551 | 79246564 |  |  |  |  |  |  |  | + |
| ZBTB42 | 100128927 | 14 | 104800597 | 104804712 |  |  |  |  |  | + |  |  |
| ZHX2 | 22882 | 8 | 122781349 | 122974515 |  |  |  |  |  |  |  | + |
| ZNF259 | 8882 | 11 | 116649276 | 116658739 | + |  |  |  |  |  |  |  |
| ZNF648 | 127665 | 1 | 182023705 | 182030847 |  |  | + |  |  | + |  |  |
| ZNF664 | 144348 | 12 | 124457762 | 124499986 |  |  | + |  |  |  |  |  |
|  |  |  |  |  |  |  |  |  |  |  |  |  |

Chr, chromosome; Start/Stop, coordinate of chromosome start and stop of the genes/loci. This gene list includes genes associated with blood lipid concentrations (Kathiresan *et al.*, 2008; Willer *et al.*, 2008; Teslovich *et al* 2010; Brouwers *et al* 2012; Lange *et al* 2015), genes in various lipid metabolism related pathways (*NCBI Biosystems BSID 106158, 106156, 106111, 198852, 194385, 198887, 160976, 160977, 106157, 106159 and 119544), genes affected by KLF4, a master regulator of gene expression in adipose tissue (Small *et al.*, 2011), and other potential candidates from personal communications.

References:

Kathiresan S, Melander O, Guiducci C, Surti A, Burtt NP, Rieder MJ, et al. Six new loci associated with blood low-density lipoprotein cholesterol, high-density lipoprotein cholesterol or triglycerides in humans. Nature Genetics. 2008;40(2):189-97.

Willer CJ, Sanna S, Jackson AU, Scuteri A, Bonnycastle LL, Clarke R, et al. Newly identified loci that influence lipid concentrations and risk of coronary artery disease. Nature Genetics. 2008;40(2):161-9.

Teslovich TM, Musunuru K, Smith AV, Edmondson AC, Stylianou IM, Koseki M, et al. Biological, clinical and population relevance of 95 loci for blood lipids. Nature. 2010;466(7307):707-13.

Small KS, Hedman AK, Grundberg E, Nica AC, Thorleifsson G, et al. Identification of an imprinted master trans regulator at the KLF14 locus related to multiple metabolic phenotypes. Nature Genetics. 2011.

Brouwers MCGJ, van Greevenbroek MMJ, Stehouwer CDA, de Graaf J, Stalenhoef AFH. The genetics of familial combined hyperlipidaemia. Nature reviews Endocrinology. 2012;8(6):352-62.

Lange LA, Willer CJ, Rich SS. Recent developments in genome and exome-wide analyses of plasma lipids. Current opinion in lipidology. 2015;26(2):96-102.
