# Supplementary material for: Three patients with homozygous familial hypercholesterolemia: Genomic sequencing and kindred analysis
Source: Mol Genet Genomic Med. 2019 Oct 16;7(12):e1007. doi: 10.1002/mgg3.1007 (PMC6900368; doi:10.1002/mgg3.1007)
Supplement: Supplementary file 2 [file MGG3-7-e1007-s002.docx]

**Ancestry determination for the *LDLR* 3kb exon 1 deletion**

To ascertain the ancestral origin of the *LDLR* exon 1 deletion, we restricted our analysis to the entire phased block spanning the deletion (chr19:9189309-12793215; Hg38 coordinates). All SNPs with QUAL “PASS” were intersected with HGDP and only overlapping SNPs were used in the following analysis. We applied binomial conditional probability to model the probability of observing the combination of genotypes flanking the *LDLR* deletion in the probands using population allele frequencies in the HGDP dataset[Alexander and others 2009]. Given the observed alternate allele count *m* of total allele count *n* in population *k*, the expected conditional probability for any SNP *s* to be derived from that population was calculated as follows:

$${{P'}_{k,s}=E}_{(f_{true}|f_{observed}=m)}=\int_{0}^{1} P_{(f_{true}=x|f_{observe}=m)}*x dx=\int_{0}^{1} \frac{P_{(f_{true}=x and f_{observe}=m)}}{P_{f_{observe}=m}}*x dx=\frac{\int_{0}^{1} P_{\left( f_{true}=x and f_{observe}=m \right)}*x dx}{\int_{0}^{1} P_{\left( f_{true}=x and f_{observe}=m \right)} dx}=\frac{Beta\left( m+2,n-m+1 \right)}{Beta\left( m+1,n-m+1 \right)}=\frac{m+1}{n+2}$$

Since all tri-allelic sites were removed, the genotype of SNPs from this phased block could either be 0 (reference allele) or 1 (alternate allele). Thus, given patient’s SNP α, the expected conditional probability *P_s,k_* of an allele derived from population k matching α was as follows:

$$P_{s,k|\alpha=0}=1-{P'}_{s,k} (when the patient^{'}s allele is 0)$$

$$P_{s,k|\alpha=1}={P'}_{s,k} (when the patient^{'}s allele is 1)$$

The *P_s,k_* of all European populations and all SNPs were calculated to generate a binomial conditional probability matrix with K columns and N rows where K is the total number of populations and N is the total number of SNPs in this phased block. A score denoting the logistic probability of this entire phased block to be derived from any European population k was then computed across all SNPs as follows:

$$\log P_{overall}=\log\left( \coprod_{1\leq k\leq N}^{N} P_{s,k} \right)=\sum_{1\leq k\leq N}^{N} \log P_{s,k}$$

In determining the ancestral origin of the *LDLR* deletion allele, it is important to note that the small number of sample sizes for each European population was a major limiting factor in our analysis. Our approach was informative in that it was able to identify the most likely ancestry of this particular genomic locus among the reference populations. However, populations not represented by the database were not included in the current analysis.

**References**:

Alexander DH, Novembre J, Lange K. 2009. Fast model-based estimation of ancestry in unrelated individuals. Genome Research 19(9):1655-1664.
